# Transcriptomic and Functional Analyses Reveal That *PpGLK1* Regulates Chloroplast Development in Peach (*Prunus persica*)

**DOI:** 10.3389/fpls.2018.00034

**Published:** 2018-01-26

**Authors:** Min Chen, Xiao Liu, Shenghui Jiang, Binbin Wen, Chao Yang, Wei Xiao, Xiling Fu, Dongmei Li, Xiude Chen, Dongsheng Gao, Ling Li

**Affiliations:** ^1^College of Horticulture Science and Engineering, Shandong Agricultural University, Tai'an, China; ^2^State Key Laboratory of Crop Biology, Shandong Agricultural University, Tai'an, China

**Keywords:** chloroplast development, chlorophyll, GLK transcription factor, peach, PpARF5

## Abstract

Peach is an ideal species for fruit tree research because of its small, fully sequenced genome. Chloroplast development is dependent on the tight cooperation between the nuclear and plastid genomes, and is regulated by GLK transcription factors. In this work, the pigment content was monitored and the chloroplast-to-chromoplast conversion during the fruit ripening was visualized by transmission electron microscopy. Localization and expression analyses showed that PpGLK1 was located in the nucleus and expressed mainly in the leaves and fruit skin. A transcriptome analysis showed that *PpGLK1* and its target genes were significantly differentially expressed in ripening peach fruit skin. *PpGLK1* silencing affected chlorophyll accumulation in peach leaves and fruits. Overexpression of *PpGLK1* rescued the phenotypes of the *Arabidopsis Atglk1Atglk2* double mutant and the tomato *uniform ripening* mutant. The results of a yeast two-hybrid analysis showed that PpGLK1 is autoactivated and that PpGLK1 (301-542 a.a.) interacted with PpARF5. Together, our results indicate that *PpGLK1* regulates chloroplast development in green tissues in peach. Therefore, it may be a promising target gene for improving the production and quality of peach by genetic engineering and breeding approaches.

## Introduction

Peach (*Prunus persica*), an economically important diploid tree crop that originated in China, is one of the most highly genetically characterized deciduous trees in fruit and forest tree research (Verde et al., 2013). Photosynthesis converts light energy into electrochemical energy, and is the source of all organic molecules in plants (Alric and Johnson, 2017); therefore, photosynthesis is a target to increase crop productivity. Leaves are the main photosynthetic organ in plants, but most fruits have partially photosynthetic metabolism before they undergo the transition to truly heterotrophic metabolism. Pavel and Dejong (1993) showed that developing peach fruit provide 5–9% of the fruit photosynthate. Chloroplast development and chlorophyll content can affect the photosynthetic capacity of green tissues (Nadakuduti et al., 2014).

Many studies have been reported in peach. For example, *PpYUC11*, which participates in auxin biosynthesis, was identified as a candidate gene involved in the regulation of the stony hard phenotype in peach (Pan et al., 2015). Zhou et al. (2015) demonstrated that *PpNAC1* can activate *PpMYB10.1*, a key gene in blood-flesh peach. Cao et al. (2016) described 12 key agronomic traits including flesh color and non-acid fruit in peach. Su et al. (2012) documented the distinctive physiological characteristics of red and green peel during fruit maturation, and Karagiannis et al. (2016) studied the effect of altitude on peach skin quality traits using comparative physiological and proteomic analyses. However, chloroplast development and photosynthesis in green tissues of peach are not fully understood.

Regulating chloroplast development and increasing the photosynthetic capacity are alternative strategies to improve the production and quality of plants. Chloroplasts are not only the sites for photosynthesis but also play important roles in the biosynthesis of many metabolites (Lopez-Juez and Pyke, 2005). In recent years, there has been increasing interest in the genetic analysis of photosynthetic processes and chloroplast development. In tomato, a ripening-related transcription factor *ARABIDOPSIS PSEUDO RESPONSE REGULATOR 2-LIKE* (*SlAPRR2*-*LIKE*), was shown to influence pigmentation, and the overexpression of *SlAPPR2*-*Like* produced a more intense green color (Pan et al., 2013). The down-regulation of *SlARF4* causes a dark-green fruit phenotype at the pre-ripening stage (Sagar et al., 2013). The *high*-*pigment* mutants (*hp1, hp2*) had higher chlorophyll level in immature fruits than wild-type (Galpaz et al., 2008). The *Curl* (*Cu*) mutant, a dominant gain-of-function mutation of *TKN2*, showed elevated chlorophyll level and increased chloroplast numbers across the entire fruit surface (Nadakuduti et al., 2014). Cytokinin has been shown to affect chloroplast function via modulating photosynthetic performance (Cortleven and Schmülling, 2015) and activating chloroplast-related genes (Cortleven et al., 2016). In addition, inactivation of the Golden 2-like transcription factor *SlGLK2* during breeding selection affected fruit chloroplast development, resulting in light green tomato fruit with reduced sugars (Powell et al., 2012).

The GLK transcription factors were first identified in maize, and were subsequently found in *Arabidopsis*, maize, rice, sorghum, and the moss *Physcomitrella patens*. To date, land plants have been found contain 1-4 GLKs but no GLK has been found in algal genomes (Wang et al., 2013). The GLK genes contain two conserved domains: a specific GLK/C-terminal box (GCT-box) only in the GLKs of land plants; and a DNA-binding domain (DBD) (Rossini et al., 2001). In *Arabidopsis*, the two GLKs act redundantly and the double mutant *Atglk1Atglk2* exhibited a pale-green phenotype (Rossini et al., 2001; Fitter et al., 2002). In tomato, the two GLKs are functionally equivalent and only *SlGLK2* is expressed in fruit, mainly in the green shoulder region. A truncated *SlGLK2* caused the loss of the green shoulder (Powell et al., 2012). Overexpression of either *SlGLK1* or *SlGLK2* led to uniform ripening, resulting in darker green immature fruit, and improved quality of mature fruit (Cheng and Lai, 2013; Nguyen et al., 2014). In pepper, *CaGLK2* was shown to regulate chloroplast development throughout the entire fruit (Brand et al., 2014). Kobayashi et al. (2012) demonstrated that the auxin signaling pathway can regulate chloroplast development via GLKs in fruit and roots. *BZR1-1D* transgenic lines produced fruits with a dark-green shoulder region and asynchronous ripening (the *Uniform ripening* (*U*) phenotype) and up-regulated expression of *SlGLK2* (Liu et al., 2014). Using chromatin immunoprecipitation analyses, Waters et al. (2009) showed that nuclear photosynthetic genes including *Lhcb3, PpLhcb5, Lhcb4.2, Lhcb6, Lhcb2.2, Lhca1, Lhca2, Lhca3, PORA, CHLM, CHLH, PsaK, PsaD*, and *GUN4* are potential target genes of GLK in *Arabidopsis*. However, little is known about the roles of GLK in peach.

In this study, the chloroplast-to-chromoplast conversion in ripening peach fruits was observed by transmission electron microscopy (TEM). A transcriptome analysis of the immature to mature epicarp showed that *PpGLK1* and its target genes were significantly differentially expressed during fruit ripening. We tested the role of *PpGLK1* by virus-induced gene silencing (VIGS) in peach and overexpression in *Arabidopsis* and tomato. Finally, we confirmed that PpGLK1 interacts with PpARF5 in peach. In conclusion, these results confirm that *PpGLK1* regulates chloroplast development in peach. Thus, it is a candidate target gene for improving photosynthesis via genetic engineering or breeding strategies.

## Materials and methods

### Plant material

Peach trees (*Prunus persica* L.cv LuYou Tao1) were grown in a greenhouse at Shandong Agriculture University, Tai'an, China. Ten peach fruits from eight different trees were sampled from 19 to 68 DAFB (days after full bloom). They were sampled at approximately 7-day intervals at 10:00 to 11:00. The exocarp was collected, and immediately frozen in liquid N_2_, and then stored at −80°C. Part of the exocarp was directly used for TEM analysis.

### Pigment determination

The chlorophyll and total carotenoid contents in peach fruit skin were measured each week during ripening and were calculated as described previously (Lichtenthaler, 1987). To measure the anthocyanin content, 0.5 g fruit skin was ground into a powder in liquid N_2_, mixed with 5 mL cold methanol containing 0.1% HCl, and then kept at 4°C for 24 h in the dark. After this, a two-buffer assay system was used to determine anthocyanin content (Jin et al., 2016).

### Electron microscopy

Samples including fruit skin, leaves, and siliques were used for the microscopic analyses. All samples were fixed in 3.5% glutaraldehyde and washed with 0.1 M phosphate buffered saline (PBS). The samples were briefly post-fixed in 1% osmium tetroxide and dehydrated in an ascending ethanol series (10–70% ethanol). After this, the samples were subjected to endosmosis, and then imbedded and polymerized in Epon812 resin. Ultra-thin sections were cut using an LKB-V ultramicrotome and stained with uranium acetate and lead citrate. Finally, ultrastructure was examined under a JEOL-1200EX TEM (JEOL, Tokyo, Japan).

### Subcellular localization analyses

The full coding sequence of PpGLK1 without the stop codon was PCR-amplified and inserted into the pPZP211 vector. The primer was shown in Table S1. The plasmids harboring the PpGLK1-GFP and the control GFP were transformed into *Agrobacterium tumefaciens* strains GV3101 and LBA4404. *A. tumefaciens* LBA4404 was used to infect onion epidermal cells (Wang et al., 2017). Equal volumes of *A. tumefaciens* GV3101 and P19 were mixed together and used to infiltrate young tobacco leaves (Jiang et al., 2017). GFP fluorescence was detected under a Zeiss LSM880 microscope (Carl Zeiss, Oberkochen, Germany) and the pictures were analyzed using ZEN lite software (https://www.zeiss.de/corporate/home.html). The subcellular localization assays were repeated more than three times with similar results.

### RNA-seq data analysis

The *P. persica* genome annotations, downloaded from the Phytozome database (https://phytozome.jgi.doe.gov/pz/portal.html), were used as a reference. Bowtie2 v2.2.3 (Langmead and Salzberg, 2012) was used to build the genome index, and clean data were mapped to the reference genome using TopHat v2.0.12. The number of reads for each gene in the samples was counted by HTSeq v0.6.0. The expression levels of the genes in each sample were estimated as Reads Per Kilobase Million Mapped Reads. DEGseq v1.14.0 software was used to identify the DEGs between two biological replicate samples using a model based on negative binomial distribution (Wang et al., 2010). A *P*-value was assigned to each gene and adjusted by the Benjamini and Hochberg approach to control the false discovery rate. Genes with *q* ≤ 0.05 and |log2_ratio| ≥ 1 were identified as DEGs.

### Isolation of RNA and qRT-PCR analysis

Total RNA was isolated from 1 g fruit skin using an RNeasy Plus Mini Kit (Qiagen) as the manufacturer's instructions and extracted three times for each sample. Three duplicates of three total RNA were reverse-transcribed for qRT-PCR first-strand cDNA synthesis using the SuperScript III First-Strand Synthesis System (Invitrogen) in a total volume of 20 μL. The qRT-PCR mixture consisted of 10 μL SYBR Premix Ex Taq (TaKaRa Biotechnology, Dalian, China), 1 μL cDNA, and 1 μL each primer pair. The qRT-PCR conditions followed the manufacturer's instructions. The primers were designed using Beacon Designer 7 software, and produced 120–200 bp amplicons (see Table S1). The thermal cycling protocol was 10 min at 95°C, 40 cycles of 95°C for 15 s, and 1 min at 58°C for annealing and extension. The specificity was assessed by a melting curve analysis and size estimation of the amplified product. The relative expression levels of DEGs were calculated using the 2^−ΔΔ*t*^ method. All statistic analysis were performed using DPSv7.05.The *Actin* reference genes Prupe.6G163400 and Prupe. 3G205200 were used to normalize the assayed genes; Prupe.6G163400 was selected because of its stable expression during fruit development (Figure S1). The results shown are the average of three independent biological replicates repeated three times.

### Virus-induced gene silencing of *PpGLK1* in peach

A specific cDNA fragment of *PpGLK1* was amplified and inserted into pTRV2. pTRV2-PpGLK1, pTRV2, and pTRV1 were individually transformed into *A. tumefaciens* strain GV3101 for the VIGS experiments. The primer was shown in Table S1. The *A. tumefaciens* GV3101 lines containing pTRV2-PpGLK1, pTRV2, and pTRV1 were selected, incubated, resuspended to an OD_600_ of 0.8 in infiltration buffer (10 mM MgCl_2_, 10 mM MES, 150 μM acetosyringone), and then kept at room temperature without shaking for 2 h. Considering the firmness of immature peach (63 DAFB), the pTRV2-PpGLK1 mixture (pTRV1:pTRV2-PpGLK1 = 1:1, v/v) and the control mixture (pTRV1:pTRV2 = 1:1, v/v) were gently vacuum-infiltrated into peach leaves and fruits for 30 min and 1 h, respectively. The infiltrated leaves were kept in the dark at 21°C overnight and then transferred to a light growth chamber. Peach leaves and fruits were sampled and photographed at 3 and 7 d after infiltration, respectively.

### Over-expression of *PpGLK1* in arabidopsis and tomato

*PpGLK1* was introduced into the pRI101-AN vector containing a GFP tag sequence to form 35S:: PpGLK1-GFP (Wang et al., 2017). The recombinant plasmid was transformed into *A. tumefaciens* LBA4404 and GV3101. The *A. tumefaciens* cells were then used to transform the vector into *Arabidopsis* (GV3101) and tomato cotyledons (LBA4404) (Fillatti et al., 1987). In *Arabidopsis*, T1 transgenic seedlings were isolated by selection on MS solid medium containing kanamycin and then grown in a light incubator. Seeds of the T3 generation were collected for later use. Transgenic tomato plants were selected on MS solid medium containing kanamycin. Tomato seedlings were grown in a light growth chamber (16-h light/8-h dark, 27°C/19°C). Three lines were verified as transgenic plants. Fruits of the selected plants were used for further analyses.

### Y2H assay

A Yeast Two-Hybrid Library was constructed using peach fruit skin. Cloning and testing bait for autoactivation and toxicity, and two-hybrid screening using yeast mating were performed according to the Matchmaker® Gold Yeast Two-Hybrid System User Manual (Clontech, Palo Alto, CA, USA). The domain-deleted form (301-542 a.a.) of PpGLK1, recombined into the pGBKT7 vector, was used for screening and the Y2H assay. The CDS of PpARF5 was cloned into the pGADT7 vector (Clontech). These two recombinant plasmids were co-transformed into Y2H Gold yeast cells, and then the cells cultured on selective medium lacking Trp and Leu (-T/-L) at 30°C. The putative transformants were transferred to selective medium lacking Trp, Leu, His, and adenine (-Leu/-Trp/-His/-Ade) with or without X-α-gal.

### BiFC assay

The CDS without stop codon of PpGLK1 was cloned to the 35S-pSPYNE-YFP vector and the CDS without stop codon of PpARF5 was cloned to the 35S-pSPYCE-YFP vector. These two recombinant plasmids were co-transformed into *A. tumefaciens* LBA4404. The mixture (35S-PpGLK1-pSPYNE:35S-PpARF5-pSPYCE = 1:1, v/v) and the control (35S-pSPYNE:35S-pSPYCE = 1:1, v/v) were used to infect onion epidermal cells for 25–30 min. Then, the onion epidermal cells were cultured on MS solid medium for 24–48 h at 28°C. Finally, YFP fluorescence was detected at an excitation wavelength of 488 nm under a confocal laser scanning microscope (Carl Zeiss, Oberkochen, Germany).

### Pull-down assay

The pull-down assay was conducted as described by Wang et al. (2017). The PpGLK1 coding sequence was combined into the pET-32a (+) vector (Novagen, Madison, WI, USA) for His-tag fusion, and PpARF5 was cloned into the pGEX-4T-1 vector containing a GST-tag sequence. Then, both recombinant plasmids were individually transformed into *Escherichia coli* BL21 (DE3) to induce tag proteins. Subsequently, His-PpGLK1 was incubated with GST-PpARF5 or GST. After immunoprecipitation with an anti-His column, the pellet fraction was detected via immunoblotting using an anti-GST antibody.

## Results

### Pigment contents and ultrastructure analysis of peach fruit skin

**Figure 1:**
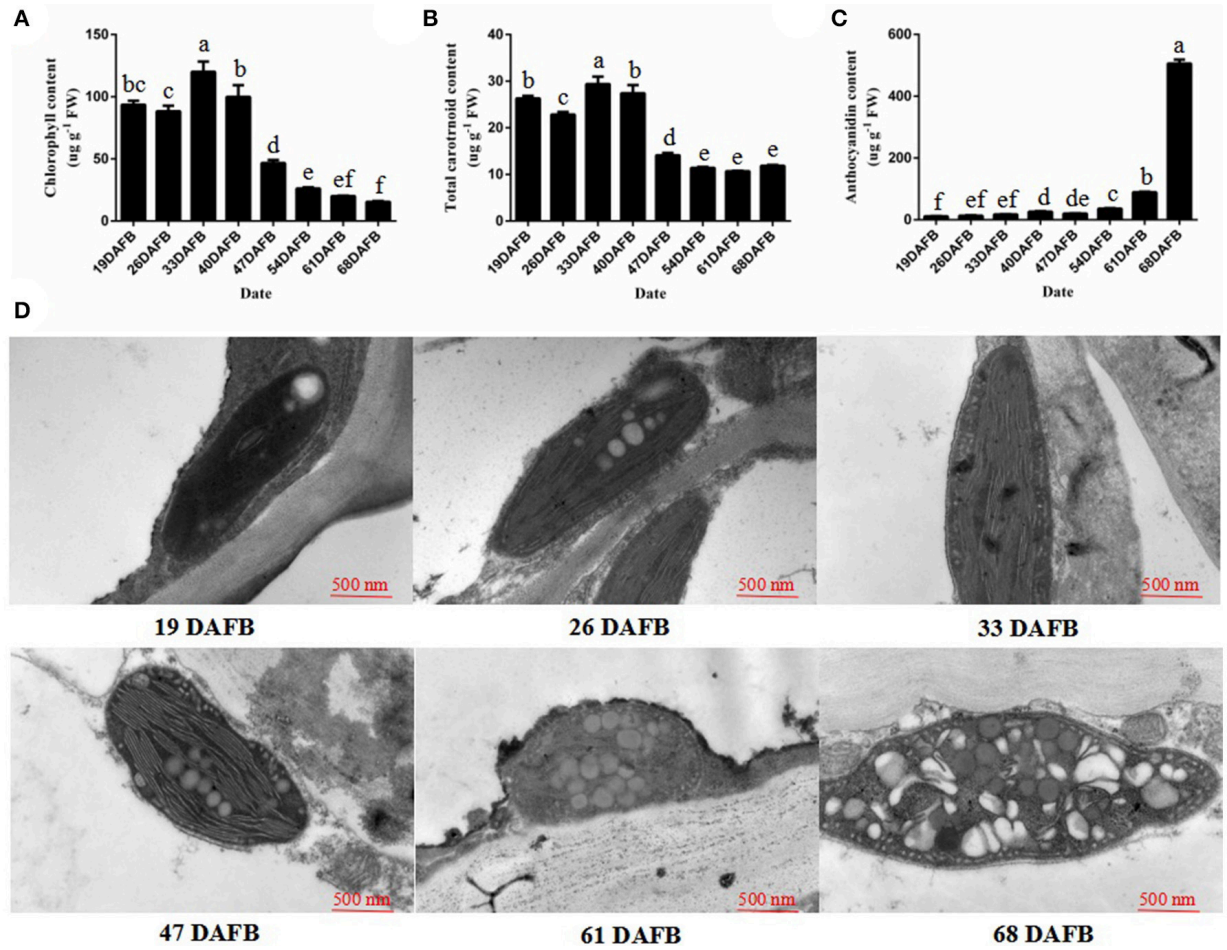
Chlorophyll, the total carotrnoid, anthocyanin contents, and chloroplast development during fruit ripening. **(A)** Chlorophyll content in peach skin during fruit development. **(B)** Total carotenoid content in peach skin during fruit development. **(C)** Anthocyanin content in peach skin during fruit development. **(D)** Transmission electron micrographs of chloroplast development in peach skin during fruit ripening. Data is presented as the means ± SD, *n* = 3. Different letters above bars denote statistical significance according to one-way ANOVA and Duncan's test (*P* < 0.05).

The fruit skin of peach fruits was analyzed at eight stages of growth to monitor the changes in pigment during fruit development. During fruit development, the chlorophyll and total carotenoid concentration decreased from 40 days after full bloom (DAFB) (Figures 1A,B). However, the anthocyanin content significantly increased, and was especially rapid from 61 DAFB onwards (Figure 1C). Then, we observed the plastids in peach fruit skin by transmission electron microscopy (TEM) at 19, 26, 33, 47, 61, and 68 DAFB. The chloroplasts in the fruit skin changed into chromoplasts as the fruit color changed (Figure 1D). Elongated chloroplasts with a clear inner structure were present in peach fruit skin at 19, 26, and 33 DAFB. Generally, these chloroplasts showed a typical arrangement of grana layers, starch grains, and parallel stromal thylakoids. Fruit coloration in peach occurred at 47 DAFB, and the chloroplast-to-chromoplast conversion caused ultrastructural changes because of the appearance of an elliptical plastid with disordered parallel stromal thylakoids and disrupted granal thylakoids. The plastoglobules were obsedved and the chloroplasts had changed into chromoplasts at 68 DAFB. Together, these results illustrated the conversion of chloroplasts into chromoplasts during peach fruit development.

### Isolation and analysis of PpGLK1

**Figure 2:**
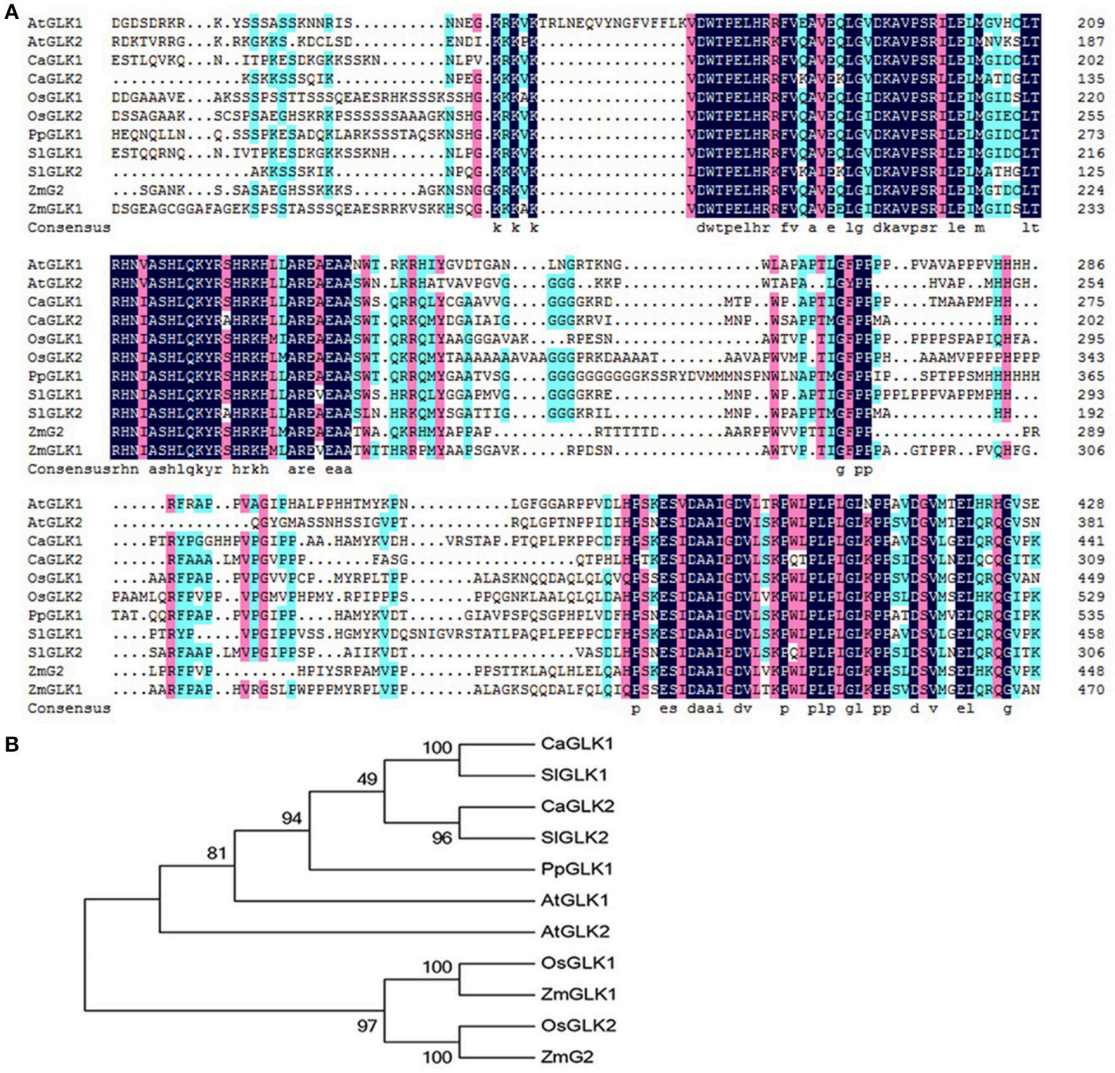
Analysis of GLKs in different species including *Arabidopsis*, tomato, pepper, rice, maize, and peach. **(A)** Multiple alignment of GLK amino acid sequences from *Arabidopsis*, tomato, pepper, rice, maize, and peach. (AtGLK1: AT2G20570; AtGLK2: AT5G44190; SlGLK1: Solyc07g053630.2.1; SlGLK2: Solyc10g008160.2.1; CaGLK1: LOC107878377; CaGLK2: LOC107845460; OsGLK1: LOC4340977; OsGLK2: LOC4326363; ZmGLK1: GRMZM2G026833; ZmG2: GRMZM2G087804; PpGLK1: Prupe.3G127700). **(B)** Phylogenetic tree analysis of GLKs from *Arabidopsis*, tomato, pepper, rice, maize, and peach. Phylogenetic tree was built using MEGA5.1 with the neighbor-joining method. Data of sequences used for phylogenetic analysis is provided in Table S4.

The peach genome contains only one GLK gene, so we named it PpGLK1. Sequence analysis showed that PpGLK1 contained 542 amino acids with a putative molecular weight of 59.17 kDa and a theoretical isoelectric point of 6.06. A multiple sequence alignment analysis demonstrated that PpGLK1 had a TEA DNA-binding domain and specific GCT-box at the C-terminal (Figure 2A).

We constructed a phylogenetic tree using the GLK amino acid sequences of *Arabidopsis thaliana, Oryza sativa, Capsicum annuum, Solanum lycopersicum*, and *Zea mays* (Figure 2B). In the phylogenetic tree, PpGLK1 was most closely related to AtGLK1 and SlGLK2. Generally, orthologs that cluster together are likely to have similar functions (Tatusov et al., 1997).

### Subcellular localization and tissue-specific expression of *PpGLK1*

**Figure 3:**
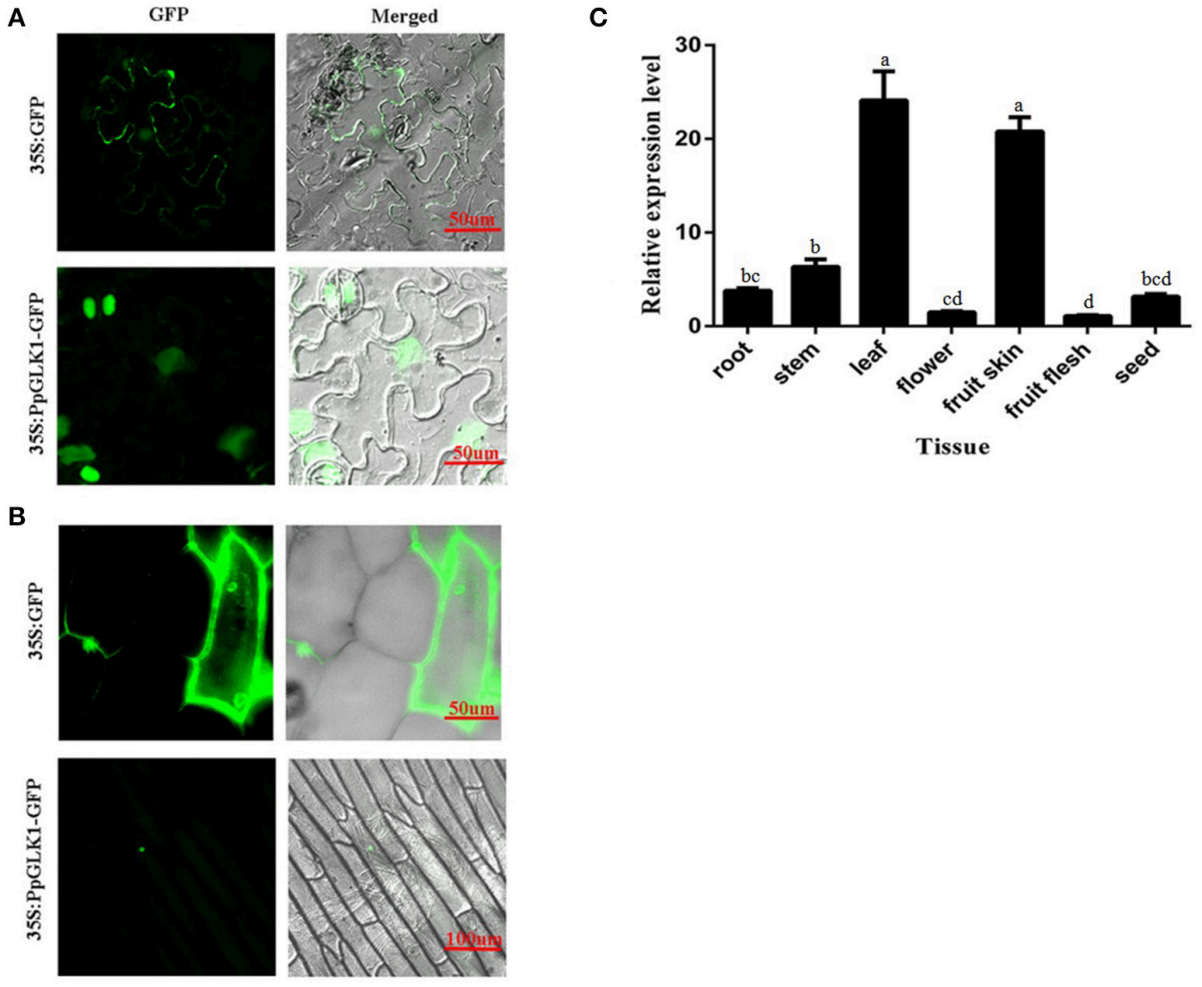
Subcellular localization and tissue-specific expression of PpGLK1. **(A)** Subcellular localization of *PpGLK1* in tobacco lower epidermal cells. Fluorescence, and merged images are shown (left to right). **(B)** Subcellular localization of PpGLK1 in onion epidermal cells. **(C)** Expression patterns of *PpGLK1* in different tissues including root, stem, leaf, flower, fruit skin, flesh, seed. Data is presented as the means ± SD, *n* = 3. Different letters above bars denote statistical significance according to one-way ANOVA and Duncan's test (*P* < 0.05).

The subcellular localization of PpGLK1 was predicted as the nucleus by WoLFPSORT (http://www.genscript.com/tools/wolf-psort). To verify the accuracy of this prediction, a fusion protein PpGLK1-GFP and a control protein GFP driven by the CaMV 35S promoter were generated and then transiently expressed in tobacco leaves and onion epidermal cells. Confocal imaging showed that the fluorescence of the control was diffuse throughout the whole cell in the tobacco leaves (Figure 3A) and the onion epidermal cells (Figure 3B). The fluorescence of PpGLK1-GFP was detected exclusively in the nucleus (Figures 3A,B), confirming the nuclear localization of PpGLK1. Next, the transcript levels of *PpGLK1* were measured in various tissues (root, stem, flower, leaf, fruit skin, fruit flesh, and seed) by qRT-PCR. There were high transcript levels of *PpGLK1* in the leaf and fruit skin, similar to the pattern of *SlGLK1* expression (Figure 3C).

### Transcriptome analysis

**Figure 4:**
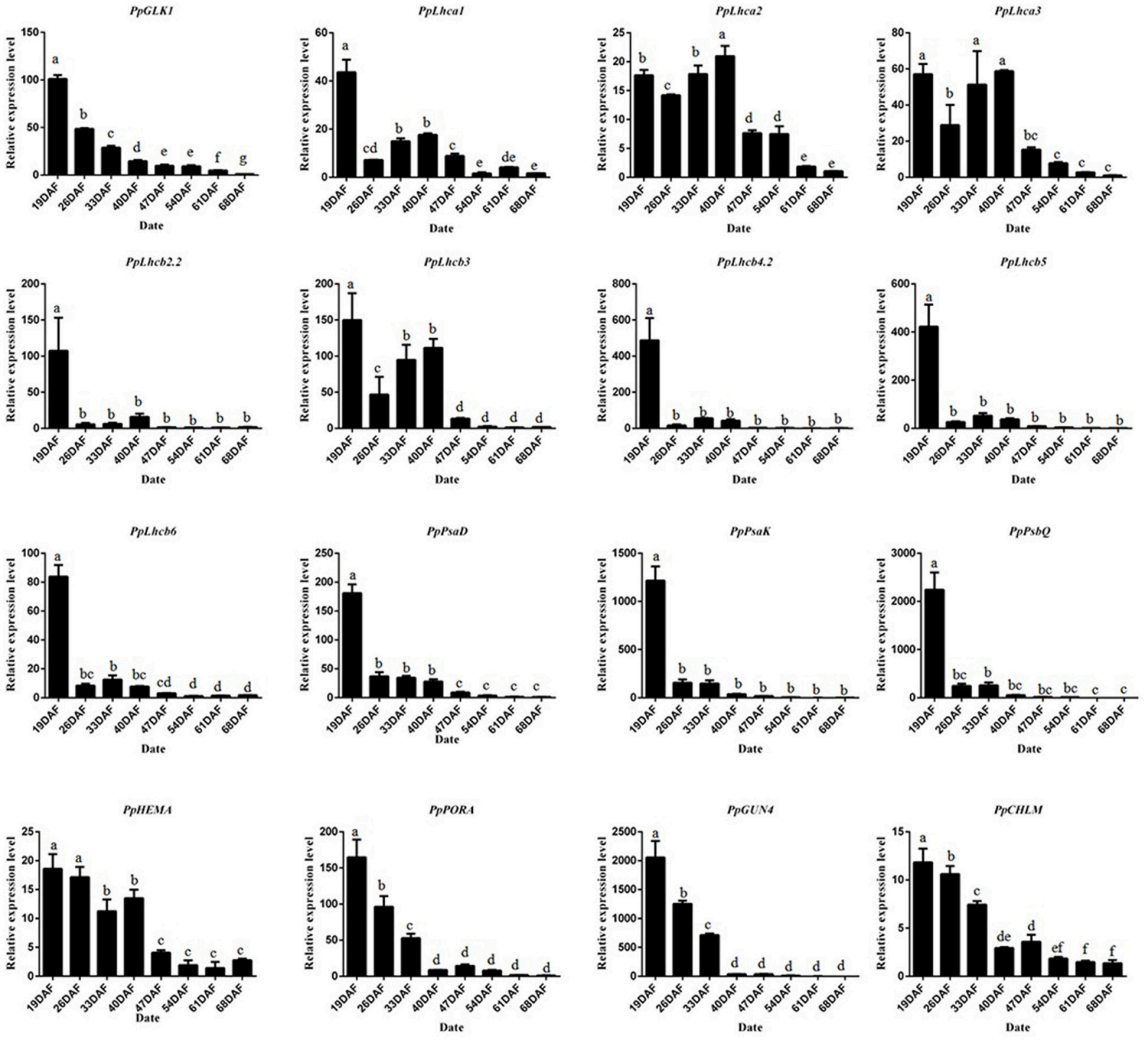
Expression analysis of *PpGLK1* and its target genes in fruit skin of 19–68 DAFB. Data is presented as the means ± SD, *n* = 3. Different letters above bars denote statistical significance according to one-way ANOVA and Duncan's test (*P* < 0.05).

To study the molecular basis of chloroplast development in peach fruit skin, we performed a transcriptome analysis of the epicarp at immature to mature stages. Six cDNA libraries were constructed using mRNA from 19 DAFB and 68 DAFB fruit skin samples, with three biological replicates (GenBank Accession number: SRX3469636 and SRX3469635). Significant differentially expressed genes (DEGs) were identified based on their | log_2_Ratio | ≥ 1 and *q* < 0.05 threshold. A Gene Ontology (GO) term analysis showed that large proportions DEGs were related to photosynthesis, light harvesting, chloroplast, and chlorophyll binding (Table S2). Importantly, the GLK transcription factor and its target genes such as *PpLhcb1.3, PpLhcb2*.2, *PpLhca3, PpHEMA, PpGUN4* all showed significant differences in expression during ripening, consistent with the changes in chlorophyll content. When gene transcript levels were determined using an alternative methodology, qRT-PCR, the results were consistent with those of the transcriptome analysis (Figure 4). The transcript levels of *PpGLK1* and its target genes decreased during fruit development. Based on these results, we concluded that *PpGLK1* may play an important role in chlorophyll biosynthesis and chloroplast development.

### Virus-induced gene silencing of *PpGLK1* influences its target genes and chlorophyll content in peach

**Figure 5:**
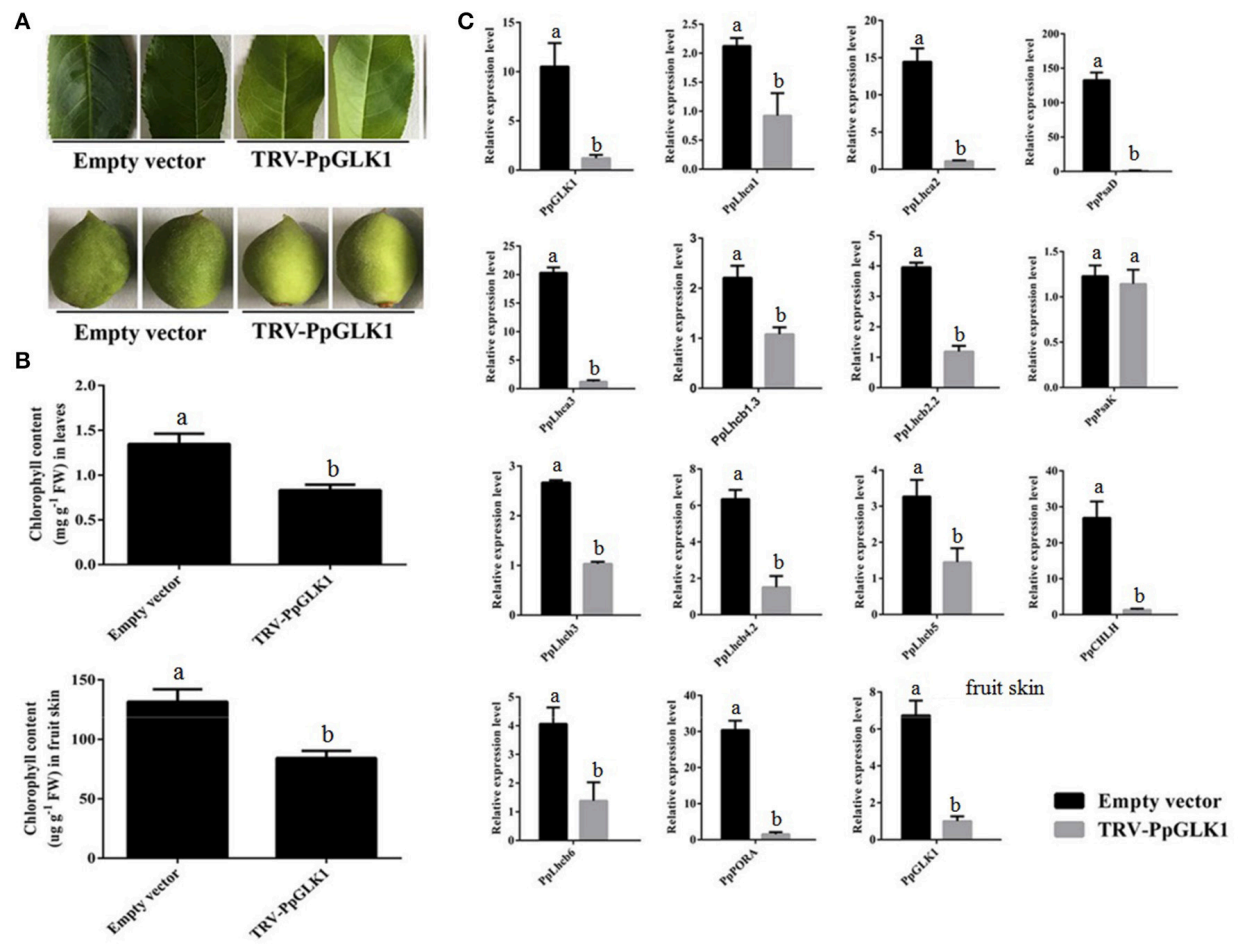
Pale-green phenotype of peach after *PpGLK1* silencing. **(A)** Phenotypes of leaves and fruits of *PpGLK1*-silenced peach. **(B)** Chlorophyll contents in leaves and fruits of *PpGLK1*-silenced and control peach. **(C)** Transcript levels of *PpGLK1* and its target genes in leaves and fruits of *PpGLK1*-silenced and control peach. Data is presented as the means ± SD, *n* = 3. Different letters above bars denote statistical significance according to one-way ANOVA and Duncan's test (*P* < 0.05).

Next, we used VIGS to eliminate *PpGLK1* expression in peach. We selected a specific cDNA fragment of the *PpGLK1* gene and introduced it into the pTRV2 vector to produce pTRV2-PpGLK1. pTRV2 was used as the control. Both vectors were infiltrated into peach fruit and leaves along with pTRV1 for VIGS. Mature leaves and immature fruit of peach were selected to validate the effect of *PpGLK1* silencing. In mature leaves, the infiltration sites on the leaves became pale green by 3 days after transformation with pTRV1 and pTRV2-PpGLK1, whereas no clear phenotype was observed after transformation with the control vector (Figure 5A). Further analyses showed that chlorophyll content at the infiltration sites of pTRV1 and pTRV2-PpGLK1 was 0.73 mg g^−1^, much lower than that at sites infiltrated with the control vector (1.41 mg g^−1^; Figure 5B). The chlorophyll content was 1.52 mg g^−1^ at non-infiltrated sites. At 3 days after transformation, the transcript level of *PpGLK1* at sites infiltrated with pTRV1 and pTRV2-PpGLK1 was 91.20% lower than that at non-infiltrated sites (Figure 5C).

Waters et al. (2009) demonstrated that GLK can bind to the promoter of many nuclear photosynthetic genes including *Lhcb3, Lhcb5, Lhcb4.2, PpLhcb6, Lhcb2.2, Lhcb1.3, Lhca1, Lhca2, Lhca3, PORA, CHLM, CHLH, PsaK, PsaD*, and *GUN4*. The transcript levels of these target genes were clearly decreased at sites infiltrated with pTRV1 and pTRV2-PpGLK1 (Figure 5C). There were no significant differences in the transcript levels of each of these genes between sites infiltrated with the control vector and uninfected leaves. In the immature fruit, infiltration sites were paler than the control by 7 days after transformation with pTRV1 and pTRV2-PpGLK1 (Figure 5A) and the expression of *PpGLK1* and its target genes were down-regulated at the infiltrated sites (Figure S2). Measurements of chlorophyll content in peach fruit peel confirmed a decrease in chlorophyll content around the infiltrated sites (Figure 5B). Thus, silencing of *PpGLK1* affected the expression of its target genes and chlorophyll content in peach. Together, these results showed that *PpGLK1* can positively regulate chlorophyll accumulation in peach fruit peel.

### Phenotypic characterization of *PpGLK1-OE* lines in *arabidopsis thaliana* and tomato

**Figure 6:**
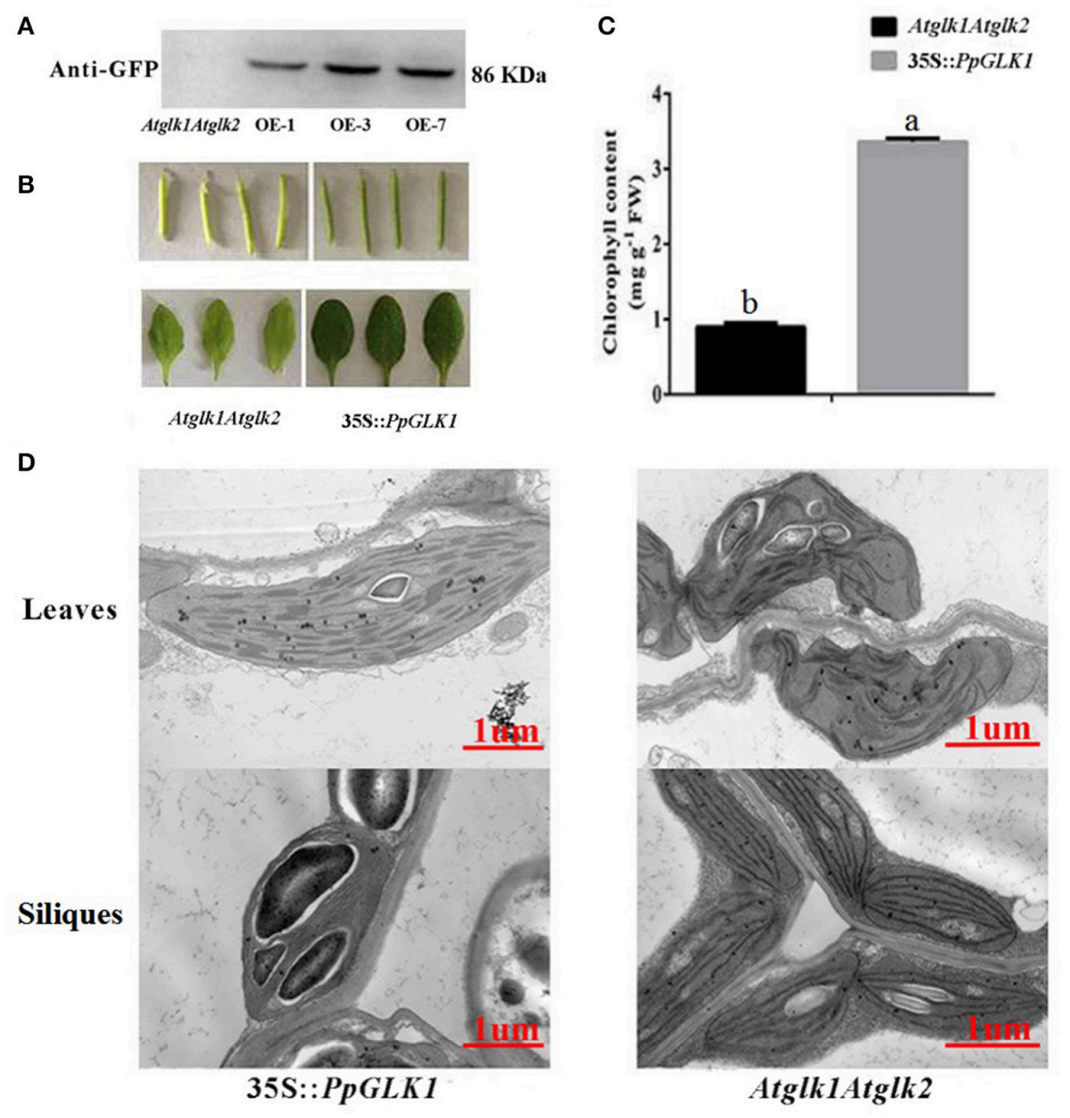
Overexpression of *PpGLK1* in *Arabidopsis* double mutant *Atglk1Atglk2*. **(A)** Western blot analysis confirming *PpGLK1* overexpression in *Atglk1Atglk2*. **(B)** Phenotype of *PpGLK1*-overexpressing *Atglk1Atglk2*. **(C)** Chlorophyll contents in *Atglk1Atglk2* overexpressing *PpGLK1* and *Atglk1Atglk2*. **(D)** Chloroplast development in siliques and leaves observed by TEM in *Atglk1Atglk2* overexpressing *PpGLK1* and *Atglk1Atglk2*. Data is presented as the means ± SD, *n* = 3. Different letters above bars denote statistical significance according to one-way ANOVA and Duncan's test (*P* < 0.05).

The GLK transcription factors have been shown to regulate chloroplast development and formation of the photosynthetic apparatus in *Arabidopsis* (Waters et al., 2009). To further verify the function of *PpGLK1*, a CaMV-35S promoter (35S::*PpGLK1*) vector was constructed and heterologously transformed into the *Arabidopsis glk1glk2* double mutant. Transgenic plants were identified by western blotting with GFP antibody (Figure 6A). Introduction of 35S::*PpGLK1* recovered the *glk1glk2* double mutant pale-green phenotype (Figure 6B); the chlorophyll content in 35S::*PpGLK1 Arabidopsis* was approximately 3.5 times that in *glk1glk2* (Figure 6C). The TEM analyses showed that there was a general increase in the number of granal thylakoids (8.9) in *PpGLK1-OE* lines, compared with *glk1glk2* leaves (1.5). The starch grains in the chloroplasts of *PpGLK1-OE* siliques were significantly larger than those of *Arabidopsis glk1glk2* mutant (Figure 6D). These results proved that *PpGLK1* could complement the chlorophyll biosynthesis phenotype of the *glk1glk2* double mutant. This complementary analysis showed that *PpGLK1* maintains its function to control chloroplast development in *Arabidopsis*, like in peach.

**Figure 7:**
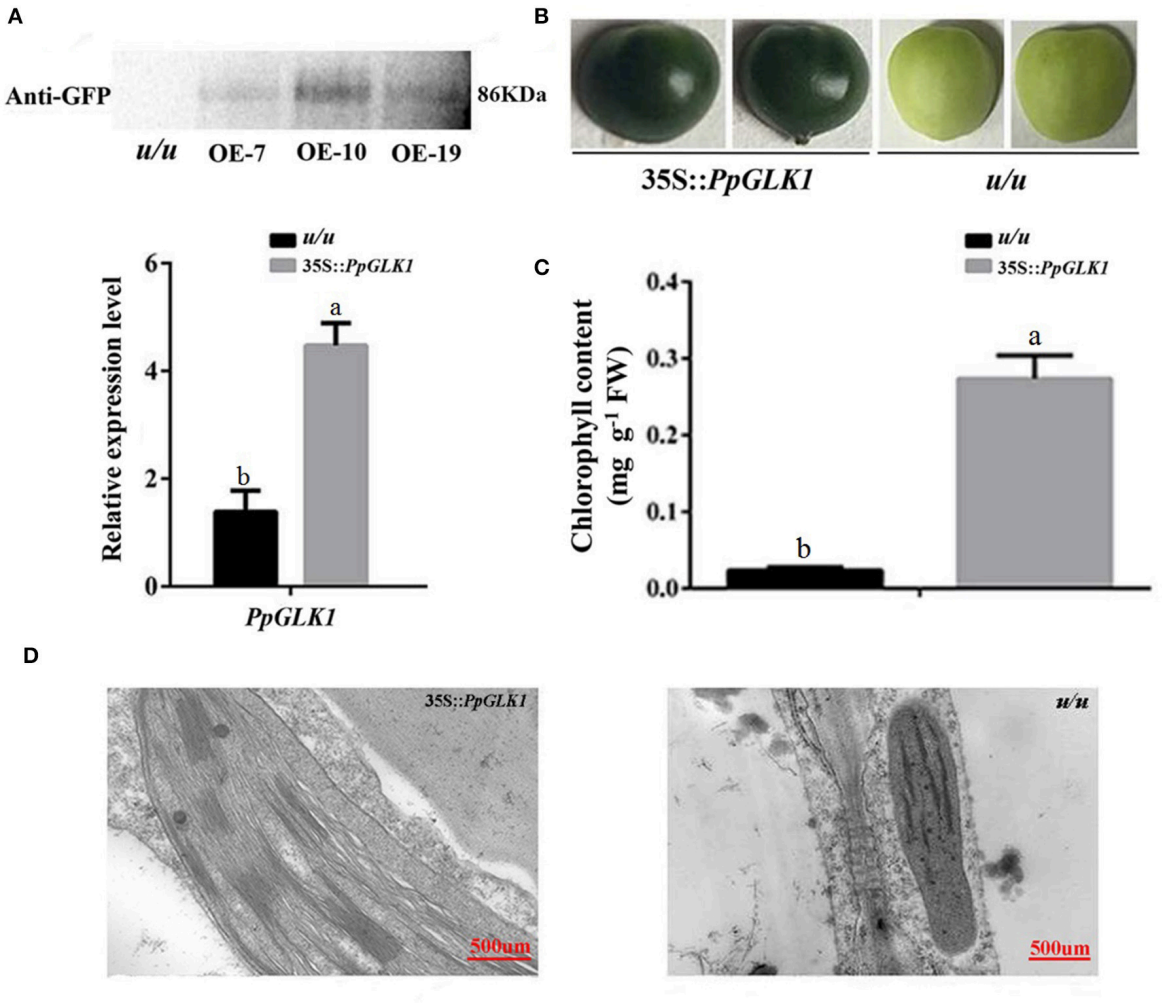
Overexpression of *PpGLK1* in *u/u* tomato. **(A)** qRT-PCR and western blot analysis confirming *PpGLK1* overexpression in *u/u* tomato. **(B)** Phenotype of *PpGLK1*-overexpressing *u/u* tomato. **(C)** Chlorophyll contents in *u/u* tomato and *PpGLK1*-overexpressing *u/u* tomato. **(D)** Chloroplast development in fruit observed by TEM in *PpGLK1*-overexpressing *u/u* tomato fruit and *u/u* tomato fruit. Data is presented as the means ± SD, *n* = 3. Different letters above bars denote statistical significance according to one-way ANOVA and Duncan's test (*P* < 0.05).

**Figure 8:**
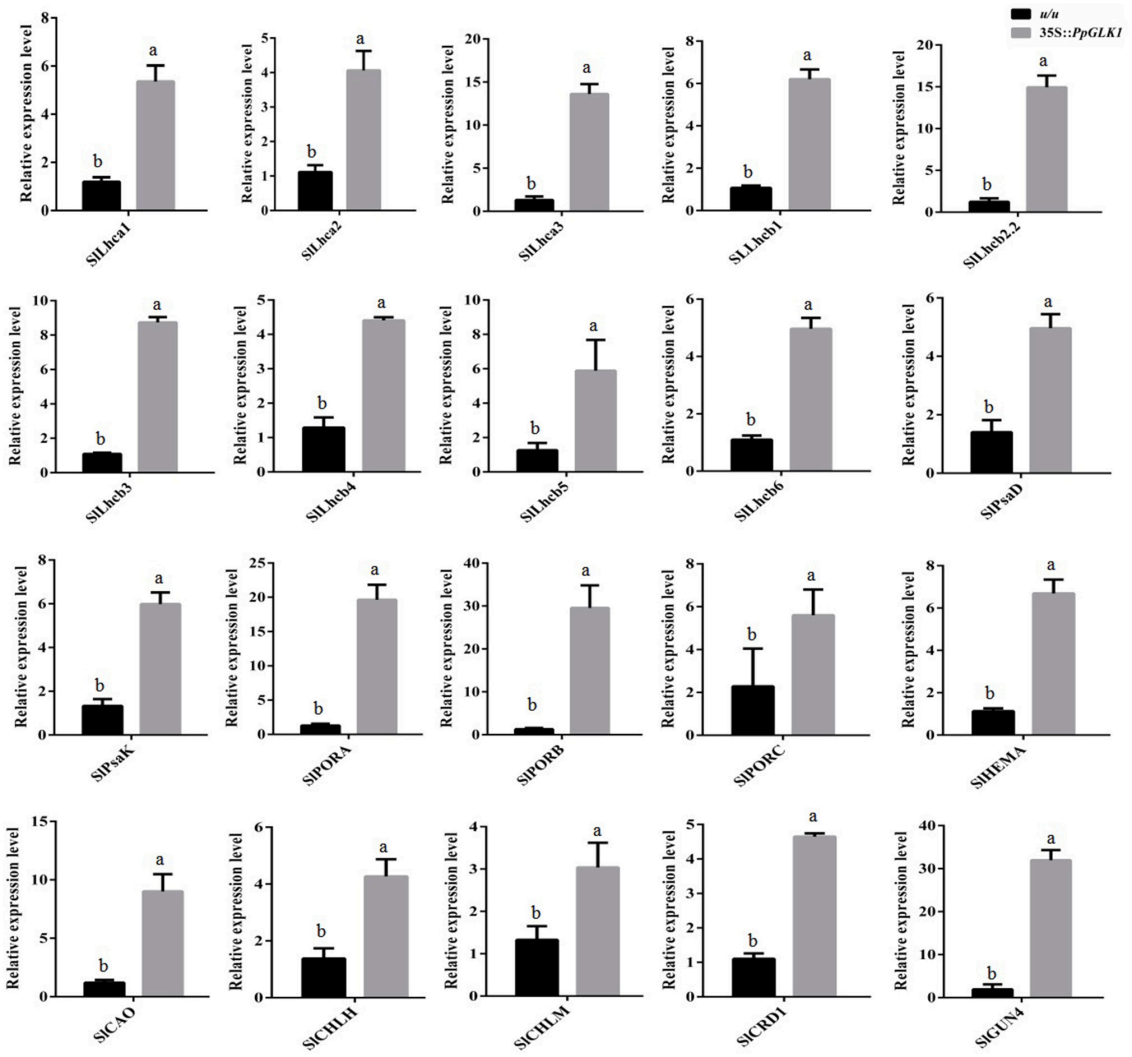
Expression patterns of *PpGLK1* target genes in *PpGLK1*-overexpressing *u/u* tomato fruit. Data is presented as the means ± SD, *n* = 3. Different letters above bars denote statistical significance according to one-way ANOVA and Duncan's test (*P* < 0.05).

As reported by Powell et al. (2012), *uniform ripening* (*u*) encodes a truncated *Slglk2* that affects fruit chloroplast development in tomato. Micro-Tom, which is homozygous for the *u* mutation (*u/u*), was selected for further experiments (Nadakuduti et al., 2014). Similar to *PpGLK1-OE* in *Arabidopsis*, Micro-Tom lines overexpressing *PpGLK1-OE* were selected by qRT-PCR and western analysis (Figure 7A). The Micro-Tom *PpGLK1-OE* lines produced uniform dark-green unripe fruit (Figure 7B). Chlorophyll content was elevated by 135-fold (Figure 7C) compared with that in untransformed Micro-Tom (*u/u*). The number and size of chloroplasts were increased and the number of thylakoids per granum was also greater in Micro-Tom *PpGLK1-OE* fruit than in *u/u* fruit (Figure 7D). Interestingly, the transcript levels of GLK target genes were also up-regulated in Micro-Tom *PpGLK1-OE* (Figure 8). Thus, the phenotypic characterization of *PpGLK1-OE* in *u/u* tomato revealed that *PpGLK1* is associated with chloroplast development and chlorophyll biosynthesis in tomato, like in peach.

### PpGLK1 can interact with PpARF5

**Figure 9:**
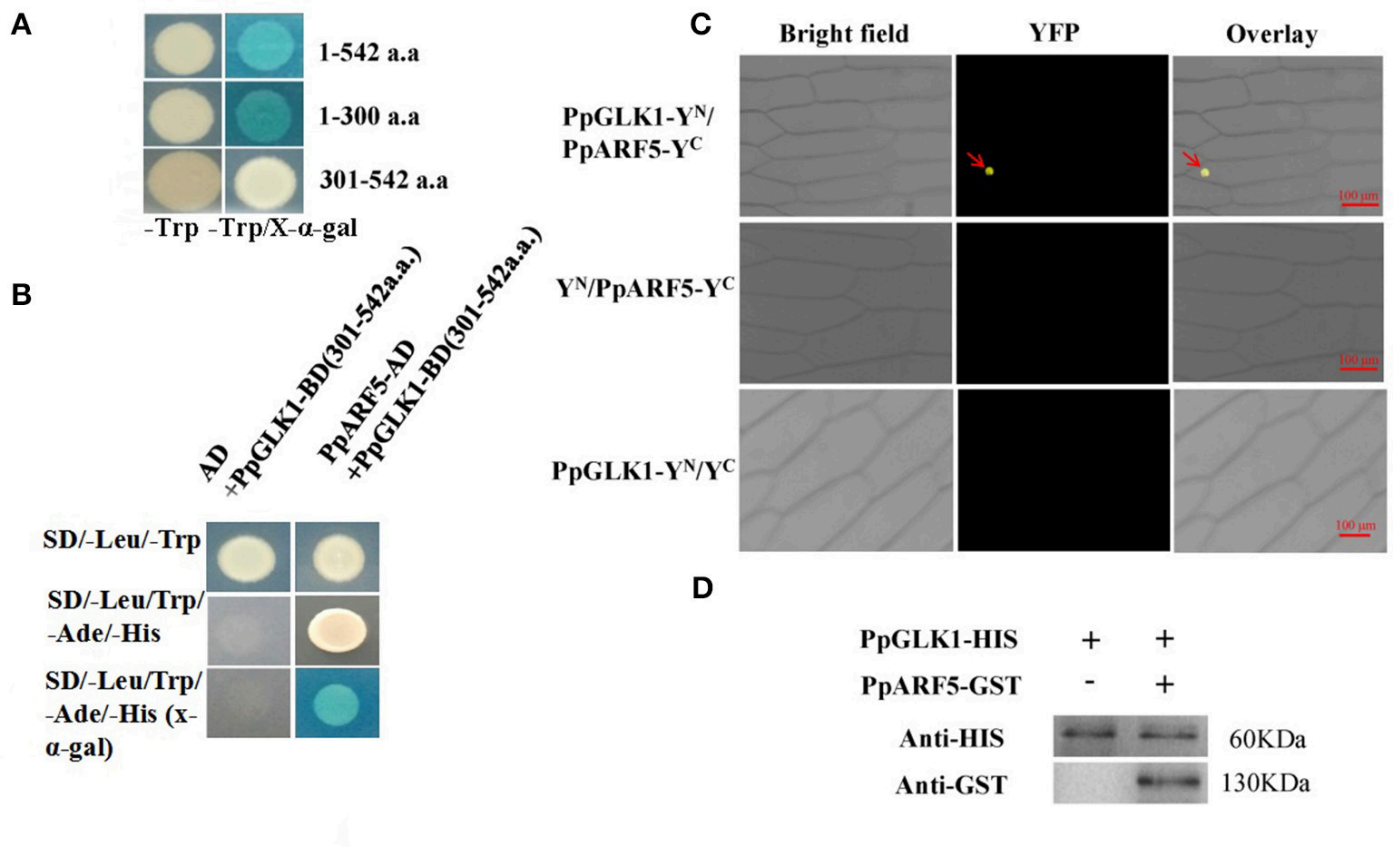
Interaction between PpGLK1 and PpARF5. **(A)** Autoactivation of PpGLK1 in yeast cells. **(B)** Interaction between PpGLK1 and PpARF5 in Y2H assay. **(C)** Interaction between PpGLK1 and PpARF5 in BiFC assay. **(D)** Interaction between PpGLK1 and PpARF5 *in Vitro* in pull-down assay.

To test bait for autoactivation and toxicity, the full-length PpGLK1 coding region fused to pGBKT7 was generated and transformed into competent Y2HGold cells; pGBKT7-53 served as the positive control. After selection on SD/-Trp, SD/-Trp/ X-a-Gal and SD/-Trp/X-a-Gal/AbA medium, we found that the expression of our bait protein was not toxic in yeast and that the PpGLK1 protein autoactivated (Figure 9A and Figure S3). To select the fragment for yeast two-hybrid (Y2H) screening, the PpGLK1 protein was cut into two fragments: 1-300 a.a. and 301-542 a.a. The 301-542 a.a. fragment did not autoactivate and was used as bait for Y2H screening (Figure 9A). In the Y2H screening, several positive colonies harbored the PpARF5 protein, which is homolog of AtARF5. In a Y2H assay, PpGLK1 (301–542) interacted with the full-length PpARF5 protein (Figure 9B and Figure S3). When this interaction was tested in a bimolecular fluorescence complementation (BiFC) analysis, PpGLK1 interacted with the full-length PpARF5 protein *in vivo* (Figure 9C). A pull-down assay confirmed the interaction between PpGLK1 and PpARF5 *in vitro* (Figure 9D).

## Discussion

Although many genes have been reported to regulate chloroplast development, only GLKs control a large number of photosynthesis-related genes (Waters et al., 2009). Thus, it is critical to elucidate the function and effects of *PpGLK1* in peach. In this study, our results showed that *PpGLK1* regulates chloroplast development in peach, leading to chlorophyll accumulation. These results indicate that *PpGLK1* is a good target for improvements in fruit quality and production via breeding or transgenic approaches.

The transition from chloroplasts into chromoplasts has been documented for ripening goji berries (Hempel et al., 2017), and was shown to be induced by fruit shading in grapefruit (Lado et al., 2015). Yang et al. (2015) demonstrated that chlorophyll content was significantly lower in a rubescent leaf color mutant of *Anthurium andraeanum* than in the wild type, and analyses of the micro- and ultra-structures in the chloroplasts showed that a chloroplast-to-chromoplast transition occurred in the leaf color mutants (Cheung et al., 1993; Yang et al., 2015). A comparative proteomic analysis showed that the proteins involved in the light reactions of photosynthesis decreased in abundance during the chloroplast-to-chromoplast transition in tomato (Barsan et al., 2012). In our study, the chlorophyll and the total carotrnoid content decreased during fruit development and analyses of ultra-structural changes in chloroplasts revealed the details of the chloroplast-to-chromoplast transition. This transition has also been documented in tomato (Harris and Spurr, 1969) and bell pepper (Spurr and Harris, 1968).

Transcriptomic profiling can reveal the patterns of gene expression over time and in different parts of the plant (Wang et al., 2009). In the present study, we added a transcriptome analysis to reveal the molecular foundation of the chloroplast-to-chromoplast transition. Interestingly, the transcript levels of *PpGLK1* and its target genes decreased during the fruit ripening in peach. In *Arabidopsis* and tomato, homologs of *PpGLK1* have been shown play an important role in chloroplast development (Waters et al., 2009; Powell et al., 2012). In addition, mutants of GLK target genes (including *CAO* and *CHLM*) exhibited pale-green phenotypes. However, whether *PpGLK1* also functions in chloroplast development in peach was yet to be confirmed. The *GLK* genes are members of the GARP family (Riechmann et al., 2000) which contains 35 genes in peach. Rossini et al. (2001) demonstrated that *GLK* genes contain two highly conserved domains. Based on these two conserved domains, we searched for and found only one *GLK* gene in peach. One explanation for this result may be that ancestral triplicated blocks are fragmentary and a recent whole-genome duplication event has not occurred in peach (Verde et al., 2013). In the phylogenetic analysis, PpGLK1 was most closely related to AtGLK1 and SlGLK2 (Figure 2B) and the multiple sequence alignment showed that they have the same functional domains (Figure 2A). All these results suggested that *PpGLK1* may have the same function in peach, tomato, and *Arabidopsis*. Overexpression of *PpGLK1* complemented the double mutant *Atglk1Atglk2 (Arabidopsis)* and *u/u* (tomato) (Figures 6B, 7B). In addition, the *PpGLK1*-complemented mutants showed increased expression of chlorophyll-related genes and increased chlorophyll content. These results provided further evidence *PpGLK1* has the same function as *AtGLK1* and *SlGLK2*, which are known to be involved in chloroplast development and chlorophyll biosynthesis. Improving the photosynthetic capacity can lead to increased carbohydrate and carotenoids contents in mature fruit (Powell et al., 2012).

The VIGS system, which is composed of TRV1 and TRV2, is a powerful tool for gene functional characterization *in vivo* (Sun et al., 2016). It has been successfully used to investigate gene function in the leaves and fruits of *Pyrus betulaefolia* (Li et al., 2017), apple (Hu et al., 2016; Jiang et al., 2017), pear (Zhai et al., 2016), and peach (Zhou et al., 2015). The *Arabidopsis* double mutant *Atglk1Atglk2* exhibited a pale-green phenotype and the tomato double mutant *u/u* produced uniformly light-green unripe fruit. In peach, silencing of *PpGLK1* decreased the chlorophyll content and led to pale-green fruit and leaves (Figures 5A,B) suggesting that GLK regulates chloroplast development in peach leaves and fruits. On the basis of our results, we concluded that PpGLK1 is positive regulator of chloroplast development like GLKs in other species.

Complex hormonal signals activate the transcription factors that regulate chloroplast development-related genes (Nakamura et al., 2009). Brassinosteroids regulate plant growth and development through *BES1* and *BZR1* transcription factors. The *bes1-D* mutant exhibited a pale-green color and exhibited a reduced chlorophyll content. Another *BES1* mutant with repressed *GLK1* and *GLK2* expression showed inhibited chloroplast development (Yu et al., 2011). Chloroplast biogenesis in greening roots was shown to depend on the combination of *HY5* and *GLKs*, which function downstream of light and auxin/cytokinin signaling pathways (Kobayashi et al., 2012). GLK proteins need partners to bind DNA (Waters et al., 2009). Tamai et al. (2002) demonstrated that AtGLK1 and AtGLK2 transactivate transcription in yeast and can interact with GBF1. In addition, AtGLK1 was shown to interact with GBF3. Our results showed that PpGLK1 can autoactivate, like AtGLK1 and AtGLK2 (Figure 9A). The results of the Y2H, pull-down, and BiFC assays all confirmed the interaction between PpGLK1 and PpARF5 (Figures 9B–D). The coordinated actions of ARF and Aux/IAA transcriptional repressors play a foremost role in auxin action. Sagar et al. (2013) showed that auxin can repress the expression of *GLK2*, and that down-regulation of *SlARF4* resulted in a dark-green fruit phenotype. It is noteworthy that the promoter of *PpGLK1* contained an auxin-responsive element, the AACGAC box (Table S3). The coordinated action of ARF and Aux/IAA transcriptional repressors play a major role in auxin action. ARF5/MP broadly regulates the expression of *Aux/IAA* genes in distinct subclades and is under negative feedback control by *Aux/IAA* genes (Krogan et al., 2014). Together, the results of this study provide further evidence for a link between auxin signaling and chloroplast development.

## Author contributions

MC, DG, and LL designed the research. MC and XL performed the experiments. MC, SJ, and XL analyzed the data. LL, DL, XC, DG, and XF contributed new models. MC, SJ wrote the manuscript. LL, CY revised the intellectual content of this manuscript. All authors read and approved the final manuscript.

### Conflict of interest statement

The authors declare that the research was conducted in the absence of any commercial or financial relationships that could be construed as a potential conflict of interest.

## Supplementary material

Figure S1 The Cq of the *Actin* genes (Prupe.6G163400 and Prupe. 3G205200).

Figure S2 Transcript levels of PpGLK1 target genes in fruit skin of *PpGLK1*-silenced and control peach.

Figure S3 The original figures of Figures 9A,B.

Table S1 The primers used in this article.

Table S2 GO term enrichment analysis of DEGs.

Table S3 The analysis of *PpGLK1* promoter.

Table S4 Data of sequences used for phylogenetic analysis.
