# Supplementary figures and images for: Transcriptomic and Functional Analyses Reveal That PpGLK1 Regulates Chloroplast Development in Peach (Prunus persica)
Source: Front Plant Sci. 2018 Jan 26;9:34. doi: 10.3389/fpls.2018.00034 (PMC5791383; doi:10.3389/fpls.2018.00034)

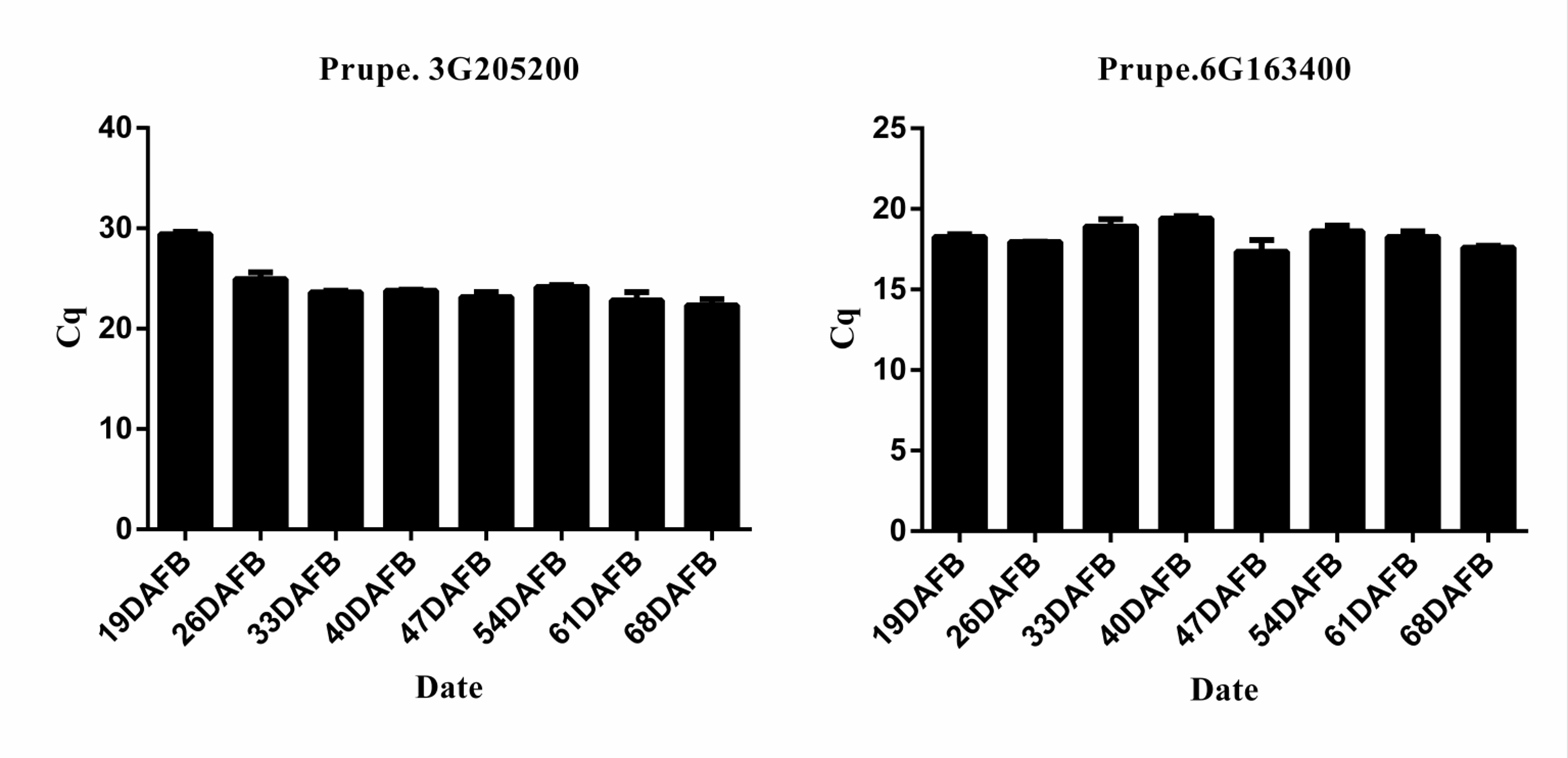

Supplement: Figure S1 — The Cq of the Actin genes (Prupe.6G163400 and Prupe. 3G205200). [file Image1.TIF]

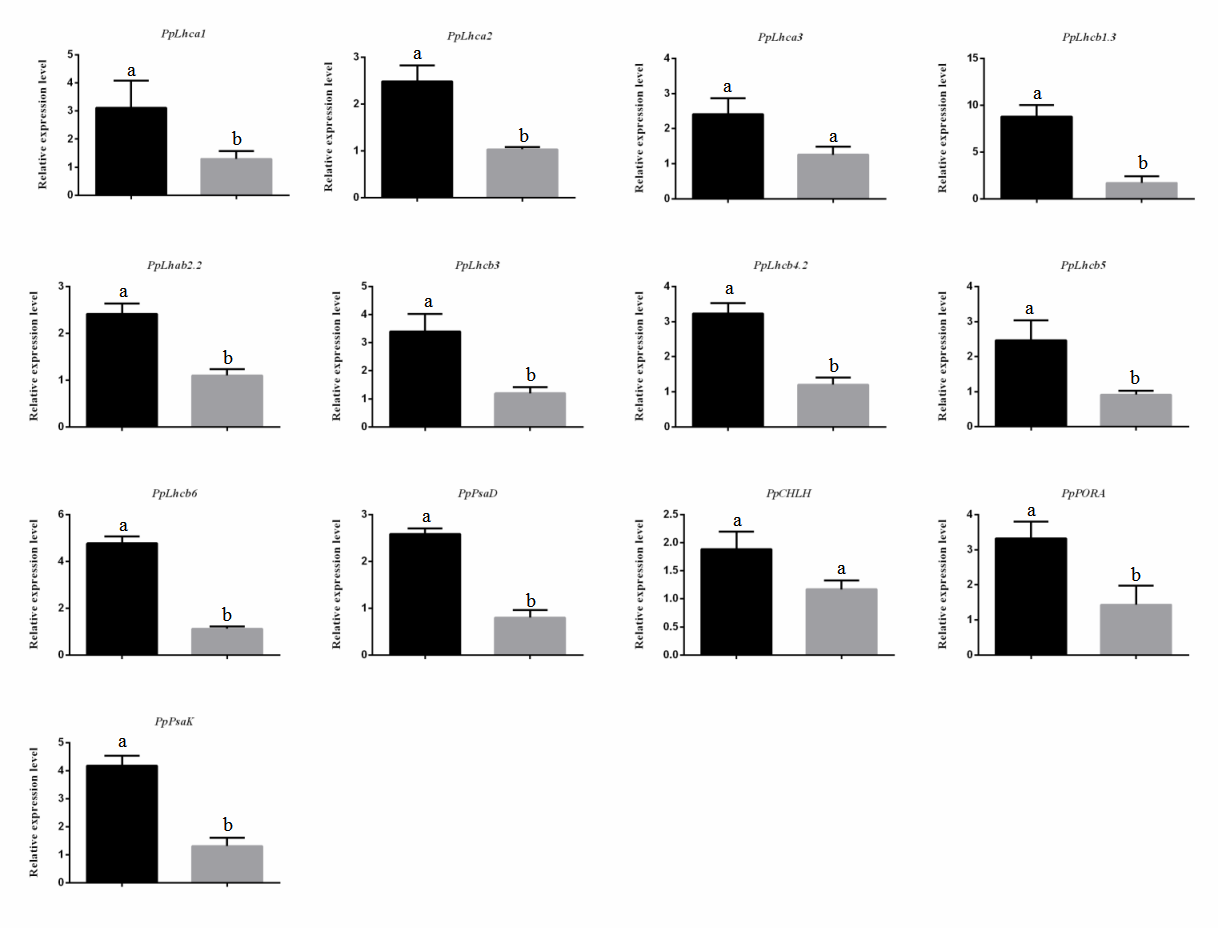

Supplement: Figure S2 — Transcript levels of PpGLK1 target genes in fruit skin of PpGLK1-silenced and control peach. [file Image2.TIF]

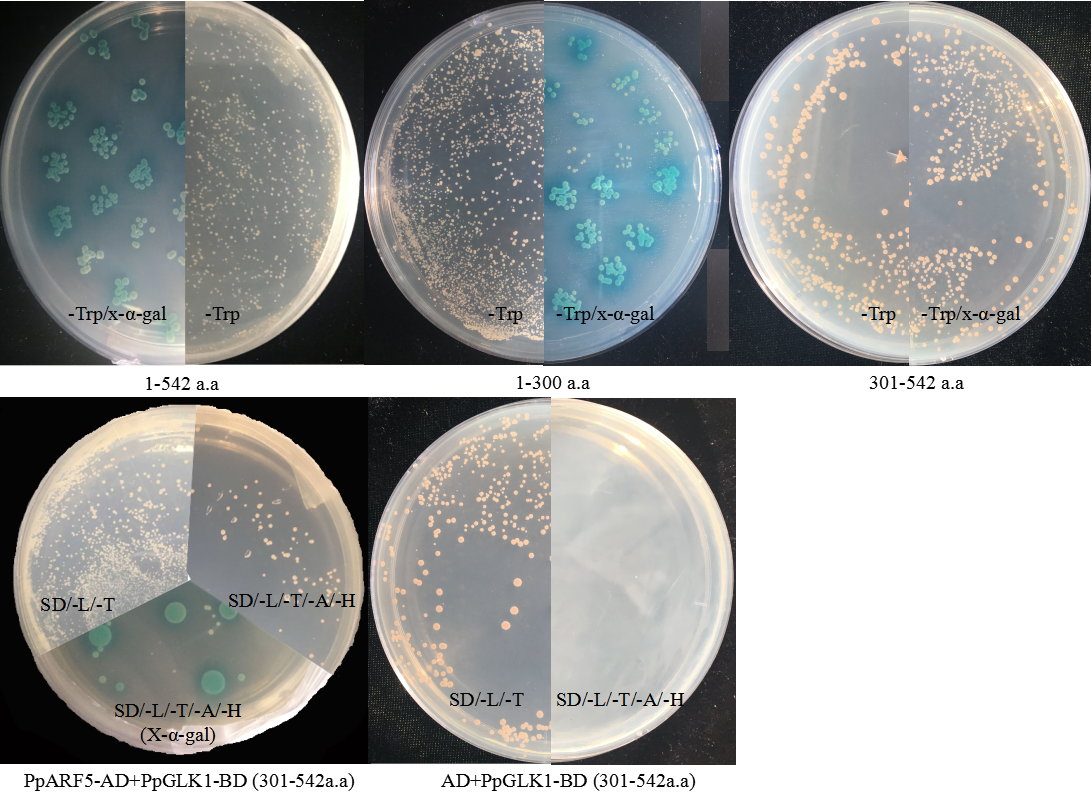

Supplement: Figure S3 — The original figures of Figures 9A,B. [file Image3.TIF]
