# Supplementary material for: Transcriptomic and Functional Analyses Reveal That PpGLK1 Regulates Chloroplast Development in Peach (Prunus persica)
Source: Front Plant Sci. 2018 Jan 26;9:34. doi: 10.3389/fpls.2018.00034 (PMC5791383; doi:10.3389/fpls.2018.00034)
Supplement: Table S1 — The primers used in this article. [file Table1.DOCX]

| **Primers for quantative RT-PCR in peach** | | | | | |
| --- | --- | --- | --- | --- | --- |
| Gene name | Forward | | Reverse | | Accession number |
| *PpCHLH* | GGTCTTGGAAGCACAGTA | | AGGTCTTGGCAGAACATT | | Prupe.7G061100 |
| *PpCHLM* | GAGGTGGTGAAGGACTAC | | TTCATCGGTCAGCATCTT | | Prupe.5G142500 |
| *PpGUN4* | TATGGCAAAGTGAGCAATG | | ATTCGTCAATGGCAGATG | | Prupe.1G193000 |
| *PpHEMA* | TTCGGCAGGAACATTAGT | | GCACCAATCACCAACATT | | Prupe.1G541100 |
| *PpLhca1* | CCTTCAGTCCTCTCATCC | | TTCTGGCACTTCTCCTAG | | Prupe.8G142700 |
| *PpLhca2* | GCTTCTTCTTCTGCCATTG | | CTGTCAAGTTCCTCACTCT | | Prupe.1G225700 |
| *PpLhca3* | CTCTGGTATCATCTTCATCCT | | TCTGCTCCTTGCTTAACA | | Prupe.3G191900 |
| *PpLhcb1.3* | GCTCAAGAACGGTAGATTG | | GACAAAGTTAGTGGCATAGG | | Prupe.4G076300 |
| *PpLhcb2.2* | GAGCATCTTGGCAATCTG | | TCACCTTCAGTTCAGCAA | | Prupe.3G201000 |
| *PpLhcb3* | TCCAGCACTCTTCTTAGAC | | CCATACCACAACTCATTTCC | | Prupe.4G190500 |
| *PpLhcb4.2* | AGTGTTGGTGATTGGGTA | | AAGCCTCTCCTTCTCTTC | | Prupe.2G248900 |
| *PpLhcb5* | GTTGTTGCTGAGGTTGTT | | TCCGTTCTTAATCTCCTTCA | | Prupe.8G001200 |
| *PpLhcb6* | AAGAACGGAGTCTACATT | | ATTAGCATAGCCACCATA | | Prupe.1G557800 |
| *PpPORA* | CCAACTGCTAAGGAACCT | | TTGAGCCAACAATGATGAG | | Prupe.4G185700 |
| *PpGLK1* | CTGCTCAATCAATCGTCAA | | TGTCCAATCCACCTTAACT | | Prupe.3G127700 |
| *PpPsaD* | GAGGAGCAGCCATTATGA | | CACCTTCTCAGGGTAAACT | | Prupe.1G011500 |
| *PpPsaK* | CACTGTGACCACTTCTCT | | CAAACCTCCCAGCAAATAG | | Prupe.4G088200 |
| *PpPsbQ* | CGGATTACCTGGAACTCTAA | | TACTCCTTCACAGCAACAA | | Prupe.4G029300 |
| *ACTIN* | CTGTGCCAATCTATGAAGGTTATG | | AGCAGTGGTGGTGAACAT | | Prupe.6G163400 |
| **Primers for quantative RT-PCR in tomato/*Arabidopsis*** | | | | | |
| Gene name | Forward | | Reverse | | Accession number |
| *SlCAO* | ATCCTCCTACAGCCACTCT | | GACACTCCATCCTTTAGCAAAC | | Solyc06g060310 |
| *SlCHLH* | GTCGTTGGCACAGGTTAT | | TCGGTCAAGGAGATTCATC | | Solyc04g015750 |
| *SlCHLM* | CTTGCTGCTATTCTATCG | | TATCTTCTTCCACCTCTG | | Solyc03g118240 |
| *SlCRD1* | ATACAGGCATCTCAAGACCAATC | | TCACATAAACCGAAAGGCAGAA | | Solyc10g077040 |
| *SlGUN4* | CCTCCTCATTCTCCAATCA | | GTTTAACCGCTGCTTCTC | | Solyc06g073290 |
| *SlHEMA* | TCAAGGTTGGTCAAGGAGTT | | AGTAGTGTGGCAGGATTCAG | | Solyc04g076870 |
| *SlLhca1* | GGCTTCTAACACATTGATGAGT | | CCGACATACTGAACCTTGAC | | Solyc05g056050 |
| *SlLhcb2.2* | GAGAAGACCGTCCTAAGT | | TAAAGCACCAAGCATAGC | | Solyc07g047850 |
| *SlLhca2* | TCCTCTTGGTCTAGCATCTGA | | GAGTCGTTGTGTCCGTGAA | | Solyc10g006230 |
| *SlLhca3* | CCACTCCACCTGTTAAGC | | ATCTTCCATTGATGACCTCTC | | Solyc12g011280 |
| *SlLhcb1.3* | TCGCTAAGAACCGTGAGT | | CCAGCCTTGAACCATACAG | | Solyc03g005780 |
| *SlLhcb3* | CAGAGCATTCTAGCAGTATTG | | TCCTTGACCTTGAGTTCC | | Solyc07g063600 |
| *SlLhcb4.2* | GGCTATGTTGGCTACTCTTG | | GAACCTCAATCCAGATCAATGT | | Solyc09g014520 |
| *SlLhcb5* | ACCAGGCAGCAATCTTGA | | AGAAGCACCACCGATGAC | | Solyc06g063370 |
| *SlLhcb6* | TTGGCACACTTCTTGGTA | | GTTCTCAGCAGTCTTGGA | | Solyc01g105030 |
| *SlPORA* | GATTGACGGTGGTGACTT | | CCCTGAATAGCCCTGTTG | | Solyc12g013710 |
| *SlPORB* | CGAGATTGTTGCTTGATGACTTGA | | TAAACCTCCCGCCATACCC | | Solyc10g006900 |
| *SlPORC* | TCTACCTCAGGACAGAAGAA | | TCGGAATGCCAAGTGATT | | Solyc07g054210 |
| *SlPsaD* | ATGGCTATGGCAACTCAA | | GTTACTGAGACGGTGGATT | | Solyc06g054260 |
| *SlPsaK* | AGCACCATCAGCAAACAG | | GTCCAAGAACAACTCCAACA | | Solyc08g006930 |
| *Actin* | TTTGCTGGTGATGATGCC | | CCTTAGGGTTGAGAGGTGCTT | | Solyc11g005330 |
| *Actin* (Arabidopsis) | CAGGATAAGGAGGGCATT | | TTTCCCAGTCAACGTCTT | | AT4G05320 |
| **Primers for PCR** | | | | | |
| PpGLK1-PRI101 | | GGATCCCAGTGGTCAAAAGTGGTGTTTG | | GTCGACTCACAAGCAACCCTTTCAAAAT | |
| **Primers for Y2H** | | | | | |
| Name | | Forward | | Reverse | |
| PpGLK1-pGBKT7 | | CATATGATGCTTATTTTATCACCTTTGAGGG | | GAATTCATCAAGCACAGGAGGGCG | |
| PpGLK1-pGBKT7(1-300a.a.) | | CATATGATGCTTATTTTATCACCTTTGAGGG | | GGATCCTCAGCTTGCTGCCTCTG | |
| PpGLK1-pGBKT7  (301-542a.a.) | | CATATGATGTGGACCCAGAGACGACA | | GGATCCTCAAGCACAGGAGGGCG | |
| PpARF5-pGADT7 | | CATATGATGATAATGGGCTCAGTTGA | | ATCGATTTAAGTACGGCCACCTTCG | |
| **Primers for subcellular localization** | | | | | |
| Name | | Forward | | Reverse | |
| PpGLK1-pPZP211 | | GGATCCCAGTGGTCAAAAGTGGTGTTTG | | GTCGACTCACAAGCAACCCTTTCAAAAT | |
| **Primers for BiFc** | | | | | |
| Name | | Forward | | Reverse | |
| PpGLK1-pSPYNE | | GGATCCCAGTGGTCAAAAGTGGTGTTTG | | GTCGACTCACAAGCAACCCTTTCAAAAT | |
| PpARF5-pSPYCE | | GGATCCATGATAATGGGCTCAGTTGA | | GTCGACAGTACGGCCACCTTCGG | |
| **Primers for pull-down** | | | | | |
| Name | | Forward | | Reverse | |
| PpGLK1-pET-32a | | GGATCCATGCTTATTTTATCACCTTTGAGGG | | GTCGACTCAAGCACAGGAGGGCG | |
| PpARF5-pGEX | | GGATCCATGATAATGGGCTCAGTTGA | | GTCGACTTAAGTACGGCCACCTTCG | |
| **Primers for VIGS** | | | | | |
| PpGLK1-pTRV2 | | TCTAGAATTATGATAATATTCGTCACCATGGTC | | GGATCCATTTTATCACCTTTGAGGGATGG | |
