# Supplementary material for: Transcriptomic and Functional Analyses Reveal That PpGLK1 Regulates Chloroplast Development in Peach (Prunus persica)
Source: Front Plant Sci. 2018 Jan 26;9:34. doi: 10.3389/fpls.2018.00034 (PMC5791383; doi:10.3389/fpls.2018.00034)
Supplement: Table S2 — GO term enrichment analysis of DEGs. [file Table2.docx]

Table S2 GO term enrichment analysis of DEGs

| Gene Ontology term | GO Term | p-value |
| --- | --- | --- |
| GO: Cellular Component |  |  |
| photosystem | GO:0009521 | 3.30E-13 |
| plasma membrane | GO:0005886 | 1.70E-11 |
| photosystem I | GO:0009522 | 3.10E-11 |
| chloroplast thylakoid | GO:0009534 | 4.00E-10 |
| plastid thylakoid | GO:0031976 | 4.00E-10 |
| chloroplast | GO:0009507 | 1.40E-09 |
| photosystem II | GO:0009523 | 2.30E-09 |
| thylakoid | GO:0009579 | 2.50E-09 |
| thylakoid membrane | GO:0042651 | 2.50E-09 |
| photosynthetic membrane | GO:0034357 | 2.50E-09 |
| plastid | GO:0009536 | 2.60E-09 |
| chloroplast thylakoid membrane | GO:0009535 | 4.60E-09 |
| plastid thylakoid membrane | GO:0055035 | 4.60E-09 |
| plastid part | GO:0044435 | 1.10E-08 |
| chloroplast part | GO:0044434 | 1.50E-08 |
| chloroplast stroma | GO:0009570 | 0.00037 |
| GO:Biological Process |  |  |
| photosynthesis | GO:0015979 | 4.90E-10 |
| photosynthesis, light harvesting | GO:0009765 | 2.10E-07 |
| chlorophyll metabolic process | GO:0015994 | 7.20E-07 |
| pigment metabolic process | GO:0042440 | 1.60E-06 |
| porphyrin-containing compound catabolic process | GO:0006787 | 1.70E-06 |
| tetrapyrrole catabolic process | GO:0033015 | 1.70E-06 |
| photosynthesis, light reaction | GO:0019684 | 1.20E-05 |
| chlorophyll catabolic process | GO:0015996 | 1.30E-05 |
| pigment catabolic process | GO:0046149 | 1.30E-05 |
| pigment biosynthetic process | GO:0046148 | 9.10E-05 |
| GO: Molecular Function |  |  |
| chlorophyll binding | GO:0016168 | 2.50E-07 |
| tetrapyrrole binding | GO:0046906 | 5.80E-06 |
