# Supplementary material for: Transcriptomic and Functional Analyses Reveal That PpGLK1 Regulates Chloroplast Development in Peach (Prunus persica)
Source: Front Plant Sci. 2018 Jan 26;9:34. doi: 10.3389/fpls.2018.00034 (PMC5791383; doi:10.3389/fpls.2018.00034)
Supplement: Table S3 — The analysis of PpGLK1 promoter. [file Table3.DOCX]

| Motif Name | Location in the Promoter | Sequence | Function |
| --- | --- | --- | --- |
| [G-Box](http://bioinformatics.psb.ugent.be/webtools/plantcare/cgi-bin/show_site_info.htpl?QWhere=ID_of_Site like 'TA~G-Box'&StartAt=0&NbRecs=10) | 784 | TCCACATGGCA | cis-acting regulatory element involved in light responsiveness |
| [TGA-element](http://bioinformatics.psb.ugent.be/webtools/plantcare/cgi-bin/show_site_info.htpl?QWhere=ID_of_Site like 'BO~TGA-element'&StartAt=0&NbRecs=10) | 336 | AACGAC | auxin-responsive element |
| [circadian](http://bioinformatics.psb.ugent.be/webtools/plantcare/cgi-bin/show_site_info.htpl?QWhere=ID_of_Site like 'LE~circadian'&StartAt=0&NbRecs=10) | 115, 1198, 800 | CAANNNNATC | cis-acting regulatory element involved in circadian control |
| TCA-element | 1291 | CAGAAAAGGA | cis-acting element involved in salicylic acid responsiveness |
| TC-rich repeats | 214, 1168, 707 | ATTTTCTTCA | cis-acting element involved in defense and stress responsiveness |
| O2-site | 1255 | GATGACATGA | cis-acting regulatory element involved in zein metabolism regulation |
| MRE | 469 | AACCTAA | MYB binding site involved in light responsiveness |
| MBS | 38, 703 | CAACTG | MYB binding site involved in drought-inducibility |
| LTR | 46 | CCGAAA | cis-acting element involved in low-temperature responsiveness |
| L-box | 1417 | AAATTAACCAAC | part of a light responsive element |
