# Supplementary material for: Transcriptomic and Functional Analyses Reveal That PpGLK1 Regulates Chloroplast Development in Peach (Prunus persica)
Source: Front Plant Sci. 2018 Jan 26;9:34. doi: 10.3389/fpls.2018.00034 (PMC5791383; doi:10.3389/fpls.2018.00034)
Supplement: Table S4 — Data of sequences used for phylogenetic analysis. [file Table4.DOCX]

>AtGLK1 AT2G20570

MLALSPATRDGCDGASEFLDTSCGFTIINPEEEEEFPDFADHGDLLDIIDFDDIFGVAGDVLPDLEIDPEILSGDFSNHMNASSTITTTSDKTDSQGETTKGSSGKGEEVVSKRDDVAAETVTYDGDSDRKRKYSSSASSKNNRISNNEGKRKVKTRLNEQVYNGFVFFLKVDWTPELHRRFVEAVEQLGVDKAVPSRILELMGVHCLTRHNVASHLQKYRSHRKHLLAREAEAANWTRKRHIYGVDTGANLNGRTKNGWLAPAPTLGFPPPPPVAVAPPPVHHHHFRPLHVWGHPTVDQSIMPHVWPKHLPPPSTAMPNPPFWVSDSPYWHPMHNGTTPYLPTVATRFRAPPVAGIPHALPPHHTMYKPNLGFGGARPPVDLHPSKESVDAAIGDVLTRPWLPLPLGLNPPAVDGVMTELHRHGVSEVPPTASCA

>AtGLK2 AT5G44190

MLTVSPAPVLIGNNSKDTYMAADFADFTTEDLPDFTTVGDFSDDLLDGIDYYDDLFIGFDGDDVLPDLEIDSEILGEYSGSGRDEEQEMEGNTSTASETSERDVGVCKQEGGGGGDGGFRDKTVRRGKRKGKKSKDCLSDENDIKKKPKVDWTPELHRKFVQAVEQLGVDKAVPSRILEIMNVKSLTRHNVASHLQKYRSHRKHLLAREAEAASWNLRRHATVAVPGVGGGGKKPWTAPALGYPPHVAPMHHGHFRPLHVWGHPTWPKHKPNTPASAHRTYPMPAIAAAPASWPGHPPYWHQQPLYPQGYGMASSNHSSIGVPTRQLGPTNPPIDIHPSNESIDAAIGDVISKPWLPLPLGLKPPSVDGVMTELQRQGVSNVPPLP

>CaGLK1 LOC107878377

MLAVSPLSNTTARDDNTMESFGIGGGVDFPDFVGENLLDSIDFDDLFVGINDGDVLPDLEMDTEILAEFSVSSGDESDVNNYSSSNKTTFISTATKKVERKDETERAASDVGSGLTSLNQGEEIVSTQKSEESTLQVKQNITPKESDKGKKSSKNNLPVKRKVKVDWTPELHRRFVQAVEQLGVDKAVPSRILEIMGIDCLTRHNIASHLQKYRSHRKHLLAREAEAASWSQRRQLYCGAAVVGGGGGKRDMTPWPAPTIGFPPPPTMAAPMPHHFRPLHVWGHPSVDQSYMHMWPKHLAPSPSPQHPSPAWAPPPHLHPPPPSDPSFWHPHHQRVPNPLTPGTPYFPAPIAPTRYPGGHHPVPGIPPAAHAMYKVDHVRSTAPPTQPLPKPPCDFHPSKESIDAAIGDVLSKPWLPLPLGLKPPAVDSVLGELQRQGVPKIPPTCA

>CaGLK2 LOC107845460

MMLVVSTPLSYKNERGNYDLFQDFPDGNLIDTIDFDDFFEGINDGDLLQNLKILDEFDISKNNTTTNLNVKTKSKENDKSKKSSSQIKNPEGKKKVKVDWTPELHRRFVKAVEKLGVDKAVPSRLLELMATDGLTRHNIASHLQKYRAHRKHLLAREAEAASWTQRKQMYDGAIAIGGGGKRVIMNPWSAPPTMGFPPMAHHIRPLHVWGHPYVNNSFWHPHYQGVPNSLAPGTPCFPSPTRFAAALMVPGVPPPFASGQTPHLHPTKESIDAAIEDVLSKPQTPLPIGLKPPSIDSVLNELQCQGITKIPPT

>OsGLK1 LOC4340977

MLAVSPAMCPDIEDRAAVAGDAGMEVVGMSSDDMDQFDFSVDDIDFGDFFLRLEDGDVLPDLEVDPAEIFTDFEAIATSGGEGVQDQEVPTVELLAPADDVGVLDPCGDVVVGEENAAFAGAGEEKGGCNQDDDAGEANVDDGAAAVEAKSSSPSSTTSSSQEAESRHKSSSKSSHGKKKAKVDWTPELHRRFVQAVEQLGIDKAVPSRILEIMGIDSLTRHNIASHLQKYRSHRKHMIAREAEAASWTQRRQIYAAGGGAVAKRPESNAWTVPTIGFPPPPPPPPSPAPIQHFARPLHVWGHPTMDPSRVPVWPPRHLVPRGPAPPWVPPPPPSDPAFWHHPYMRGPAHVPTQGTPCMAMPMPAARFPAPPVPGVVPCPMYRPLTPPALASKNQQDAQLQLQVQPSSESIDAAIGDVLSKPWLPLPLGLKPPSVDSVMGELQRQGVANVPPACG

>OsGLK2 LOC4326363

MLEVSTLRSPKADQRAGVGGHHVVGFVPAPPSPADVADEVDAFIVDDSCLLEYIDFSCCDVPFFHADDGDILPDLEVDPTELLAEFASSPDDEPPPTTSAPGPGEPAAAAGAKEDVKEDGAAAAAAAAAADYDGSPPPPRGKKKKDDEERSSSLPEEKDAKNGGGDEVLSAVTTEDSSAGAAKSCSPSAEGHSKRKPSSSSSSAAAGKNSHGKRKVKVDWTPELHRRFVQAVEQLGIDKAVPSRILELMGIECLTRHNIASHLQKYRSHRKHLMAREAEAASWTQKRQMYTAAAAAAAVAAGGGPRKDAAAATAAVAPWVMPTIGFPPPHAAAMVPPPPHPPPFCRPPLHVWGHPTAGVEPTTAAAPPPPSPHAQPPLLPVWPRHLAPPPPPLPAAWAHGHQPAPVDPAAYWQQQYNAARKWGPQAVTPGTPCMPPPLPPAAMLQRFPVPPVPGMVPHPMYRPIPPPSPPQGNKLAALQLQLDAHPSKESIDAAIGDVLVKPWLPLPLGLKPPSLDSVMSELHKQGIPKVPPAASGAAG

>SlGLK1 Solyc07g053630.2.1

MLVVSPFSNTTARDERGNEMESFAIGGGGGGGDDFPDFMGENLLDSIDFDDLFVGINDGDVLPDLEMDTEILAEFSVSSGDESDVNNYSSSNNNNTFITTAIKNVERKEEIEKTGSVSASDVGSGLTTSLNQGEEIVSTQKSEESTQQRNQNIVTPKESDKGKKSSKNHNLPGKRKVKVDWTPELHRRFVQAVEQLGVDKAVPSRILEIMGIDCLTRHNIASHLQKYRSHRKHLLAREVEAASWSHRRQLYGGAPMVGGGGGKREMNPWPAPTIGFPPPPPLPPPVAPPMPHHFRPLQVWGHPSVDQSYMHMWPKHLAPSPSPQHPSPAWAPPPHHLHPPPPLDPSFWHPHHQRVQNSLTPGTPYFPAPTRYPVPGIPPVSSHGMYKVDQSNIGVRSTATLPAQPLPEPPCDFHPSKESIDAAIGDVLSKPWLPLPLGLKPPAVDSVLGELQRQGVPKIPPTCA

>SlGLK2 Solyc10g008160.2.1

MLALSSSLSYKNERENYDLFQDFSHGNLIDTINFDDFFDEINGGDLLPDFEIFCEEPAIHGNMKSKSKEAKKSSSKIKNPQGKKKVKLDWTPELHRKFVKAIEKLGVDKAVPSRILELMATHGLTRHNIASHLQKYRAHRKHLLAREAEAASLNHRKQMYSGATTIGGGGKRILMNPWPAPPTMGFPPMAHHVRPLHVWGHPHVNNSFWHPHYQRVSNSLVPGTPCFSAPITSARFAAPLMVPGIPPSPAIIKVDTVASDLHPSNESIDAAIEDVLSKPQLPLPIGLKPPSIDSVLNELQRQGITKIPPT

>ZmG2 GRMZM2G087804

MLEVSTLRGPTSSGSKAEQHCGGGGGFVGDHHVVFPTSGDCFAMVDDNLLDYIDFSCDVPFFDADGDILPDLEVDTTELLAEFSSTPPADDLLAVAVFGADDQPAAAVAQEKPSSSLEQTCGDDKGVAVAAARRKLQTTTTTTTTEEEDSSPAGSGANKSSASAEGHSSKKKSAGKNSNGGKRKVKVDWTPELHRRFVQAVEQLGIDKAVPSRILEIMGTDCLTRHNIASHLQKYRSHRKHLMAREAEAATWAQKRHMYAPPAPRTTTTTDAARPPWVVPTTIGFPPPRFCRPLHVWGHPPPHAAAAEAAAATPMLPVWPRHLAPPRHLAPWAHPTPVDPAFWHQQYSAARKWGPQAAAVTQGTPCVPLPRFPVPHPIYSRPAMVPPPPSTTKLAQLHLELQAHPSKESIDAAIGDVLVKPWLPLPLGLKPPSLDSVMSELHKQGVPKIPPAAATTTGATG

>ZmGLK1 GRMZM2G026833

MLAVSPSPVRCADAEECGGGGASKEMEETAVGPVSDSDLDFDFTVDDIDFGDFFLRLDDGDDALPGLEVDPAEIVFADFEAIATAGGDGGVTDQEVPSVLPFADAAHIGAVDPCCGVLGEDNDAACADVEEGKGECDHADEVAAAGNNNSDSGEAGCGGAFAGEKSPSSTASSSQEAESRRKVSKKHSQGKKKAKVDWTPELHRRFVQAVEELGIDKAVPSRILEIMGIDSLTRHNIASHLQKYRSHRKHMLAREVEAATWTTHRRPMYAAPSGAVKRPDSNAWTVPTIGFPPPAGTPPRPVQHFGRPLHVWGHPSPTPAVESPRVPMWPRHLAPRAPPPPPWAPPPPADPASFWHHAYMRQGPAAHMPDQVAVTPCVAVPMAAARFPAPHVRGSLPWPPPMYRPLVPPALAGKSQQDALFQLQIQPSSESIDAAIGDVLTKPWLPLPLGLKPPSVDSVMGELQRQGVANVPQACG

>PpGLK1 Prupe.3G127700

MLILSPLRDGHHQLKEEKQLGGGGRDEGEVEGFDFNGGNSVGSAGLDFPEFSGGMGNLLDSIDFDDLFIGIHDGDVLPDLEMDSEILDFSAHRDHGDEYYHNNAKTTSSKVEDQEEEEEEEEEGKDRVRAAADSLTNATTTRNNQTYSSSSNTSTTTTSATQDQDRDGGHEEIGSKGNCNSNINNNISQIRASQHEQNQLLNQSSSPKESADQKLARKSSSTAQSKNSHGKRKVKVDWTPELHRRFVQAVEQLGVDKAVPSRILELMGIDCLTRHNIASHLQKYRSHRKHLLAREAEAASWTQRRQMYGAATVSGGGGGGGGGGGKSSRYDVMMMNSPNWLNAPTMGFPPIPSPTPPSMHHHHHHQMIRPLHVWGHPSMDQSMMHMWPKHLPHNFPSPIPPPPPPAHAWPPPPPPPSDASYWHHPPPHHQRVPNALTPGTPCFPRQLATATQQRFPAPPVPGIPPHAMYKVDTGIAVPSPQSGPHPLVDFHPSNESIDAAIGDVLSKPWLPLPLGLRPPATDSVMVELQRQGIPKIPPSCA
